# LGR5^+^ epithelial tumor stem-like cells generate a 3D-organoid model for ameloblastoma

**DOI:** 10.1038/s41419-020-2560-7

**Published:** 2020-05-07

**Authors:** Ting-Han Chang, Rabie M. Shanti, Yanfang Liang, Jincheng Zeng, Shihong Shi, Faizan Alawi, Lee Carrasco, Qunzhou Zhang, Anh D. Le

**Affiliations:** 1 Department of Oral and Maxillofacial Surgery and Pharmacology, University of Pennsylvania School of Dental Medicine, Philadelphia, PA USA; 2 Department of Oral & Maxillofacial Surgery, Penn Medicine Hospital of the University of Pennsylvania, Perelman Center for Advanced Medicine, Philadelphia, PA USA; 3 Department of Pathology, Dongguan Hospital Affiliated to Medical College of Jinan University, The Fifth People’s Hospital of Dongguan, Dongguan, 523905 China; 4 Dongguan Key Laboratory of Medical Bioactive Molecular Developmental and Translational Research, Guangdong Provincial Key Laboratory of Medical Molecular Diagnostics, Guangdong Medical University, Dongguan, 523808 China; 5 Department of Pathology, University of Pennsylvania School of Dental Medicine, Philadelphia, PA USA

**Keywords:** Mechanisms of disease, Stem-cell research

## Abstract

Ameloblastoma (AM) is a benign but locally aggressive tumor with high recurrences. Currently, underlying pathophysiology remains elusive, and radical surgery remains the most definitive treatment with severe morbidities. We have recently reported that AM harbors a subpopulation of tumor epithelial stem-like cells (AM-EpiSCs). Herein, we explored whether LGR5^+^ epithelial cells in AM possess stem-like cell properties and their potential contribution to pathogenesis and recurrence of AM. We found that LGR5 and stem cell-related genes were co-expressed in a subpopulation of AM epithelial cells both in vivo and in vitro, which were enriched under 3D-spheroid culture. As compared to LGR5^−^ counterparts, LGR5^+^ AM epithelial cells showed increased expression of various EMT- and stemness-related genes, and functionally, exhibited increased capacity to form 3D-spheroids and generate human tumor 3D organoids, which recapitulated the histopathologic features of distinct subtypes of solid AM, thus, contributing a useful human tumor platform for targeted therapeutic screening. Treatment with a selective BRAF^V600E^ inhibitor, vemurafenib, unexpectedly enriched the subpopulation of LGR5^+^ AM-EpiSCs in tumor 3D organoids, which may have explained therapeutic resistances and recurrences. These findings suggest that LGR5^+^ AM-EpiSCs play a pivotal role in pathogenesis and progression of AM and targeted inhibition of both BRAF and LGR5 potentially serves a novel nonsurgical adjuvant therapeutic approach for this aggressively benign jaw tumor.

## Introduction

Ameloblastoma (AM) is a benign but locally aggressive epithelial tumor with a high recurrent rate in the jaw bones^1^. The WHO has recently updated AM into three categories, AM (solid/multicystic type), unicystic type, and peripheral/extraosseous type, among which the solid/multicystic type accounts for around 80% of all AM cases and manifests with a high recurrence rate^2^. Currently, the ablative surgery remains the mainstay of treatment for AM, but severe morbidities associated with large jaw defects, impaired oral functions and facial esthetics that require comprehensive tissue reconstruction and oral rehabilitation significantly compromise patient quality of life and raise the overall healthcare cost^1,3^. Although about 46–82% of AM cases exhibit BRAF^V600E^ mutation^4–9^, it has no significant correlation with tumor recurrence. Clinically, the specific BRAF^V600E^ inhibitor, vemurafenib, has been utilized to treat melanoma patients, but a high drug resistance rate has been reported^10^. Currently, an active clinical trial (NCT02367859) with the combinatory use of dabrafenib (a BRAF inhibitor) and trametinib (MEK inhibitor) for treatment of AM is undergoing, but the clinical outcomes remain unknown. Further studies are necessary to delineate the mechanisms underlying AM pathogenesis that may hold promises in developing new drugs as a nonsurgical adjunctive treatment of AM.

Cancer stem cells (CSCs), or tumor-initiating cells (TICs), have the capabilities of self-renewal and differentiation into non-CSCs to repopulate the cancer mass. CSCs play important roles in tumorigenesis, progression, metastasis, therapeutic resistance, and recurrence^11,12^. In tumors, a proportion of tumor cells undergo dynamic bidirectional epithelial–mesenchymal transition (EMT) and mesenchymal–epithelial transition processes that govern the cell plasticity and are closely associated with the development of several cancer hallmarks^13^. EMT intermediate cells or hybrid cells are endowed with both epithelial and mesenchymal cell features and contribute to tumor initiation, progression, metastasis, and drug resistance^13–15^. However, much less work has been done to explore the potential role of EMT and TICs in the development of benign epithelial tumors. Recently, we have shown that AM tissues harbor a proportion of epithelial cells simultaneously expressing EMT regulatory transcription factors (TFs) such as ZEB1, Slug, and Snail as well as stem cell-related genes such as aldehyde dehydrogenase 1 (ALDH1), BMI-1, and SOX2^16^, thus supporting the notion that a subpopulation of AM epithelial cells are endowed with both EMT and stem-like cell properties (AM-EpiSCs). However, there is still lack of a putative surface marker to identify these EMT intermediate AM-EpiSCs, and their potential role in pathogenesis and progression of AM remains largely unknown.

Leucine-rich repeat-containing G-protein coupled receptor (LGR) proteins are a unique class of evolutionarily conserved seven-transmembrane receptors characterized by a large extracellular region (ectodomain) that harbors multiple imperfect copies of leucine-rich repeat protein interaction domain^17^. LGR5, a family member of LGR proteins, can activate Wnt/*β*-catenin pathway through binding with its ligands, R-spondin family (R-spondin 1–4)^17^, and has been identified as an epithelial stem cell marker in multiple developmental organs, such as the root cervical loop, taste bud, intestine, and hair follicle^17–19^. Meanwhile, LGR5 has also been reported as a putative marker for CSCs in several types of cancers, e.g. basal cell carcinoma, glioma, and gastrointestinal cancers^20–25^. Functionally, LGR5 has been shown to promote EMT process and metastasis in hepatocellular carcinoma, colon cancer, and glioma^24–26^ and to predict poor survival of glioma patients^25^. The development of odontogenic tumors, including AM, has been linked to the enamel organ, e.g., remnants of odontogenic epithelium, the migrating epithelium at the cervical loop, and lining of odontogenic cyst^1,27^. Several studies have also reported LGR5 expression in odontogenic epithelial stem cells^28–31^.

In this study, we identified a putative subpopulation of LGR5^+^ epithelial cells with stem-like cell properties characteristic of a hybrid EMT phenotype, elevated self-renewal and tumor-propagating capabilities, and resistance to a selective BRAF^V600E^ inhibitor. Importantly, we generated human tumor 3D organoids with AM epithelial cells, which recapitulated the histopathologic features of solid AM. Our findings suggest that a putative subpopulation of LGR5^+^ epithelial stem-like cells in AM (AM-EpiSCs) capable of generating tumor 3D organoids may contribute to pathogenesis of AM, and targeted inhibition of both BRAF and LGR5 potentially serves a novel nonsurgical adjuvant therapeutic approach for this aggressively benign jaw tumor.

## Results

### LGR5 was highly expressed in epithelial cells in AM tissues

**Fig. 1:**
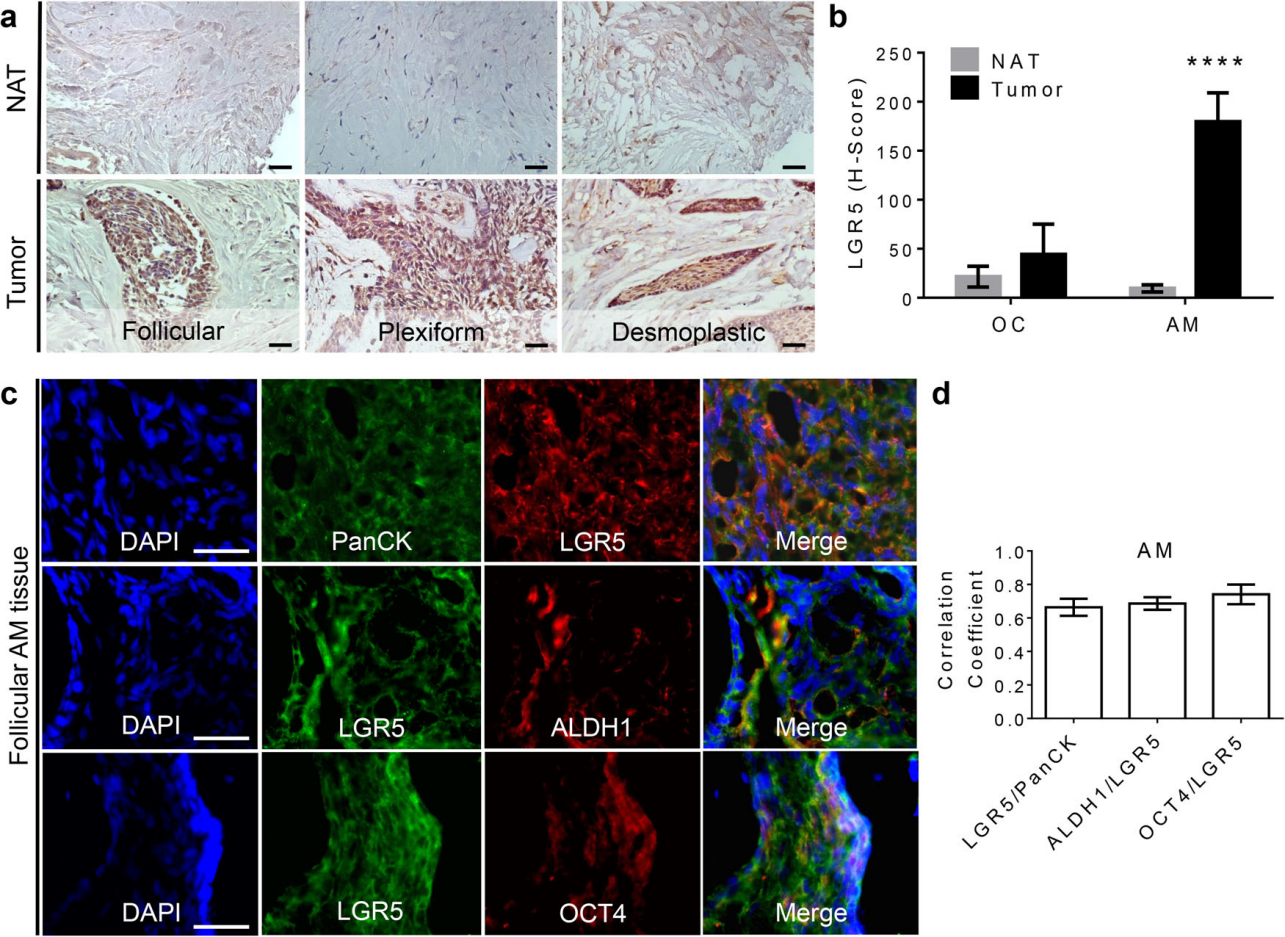
LGR5 is highly expressed in epithelial cells in AM tissues. **a** Expression of LGR5 in different histopathological types of AM (*n* = 15). Scale bars, 20 μm. NAT normal adjacent tissue (same patient). **b** The quantification of H-score of LGR5 expression in AM (*n* = 15) and odontogenic cyst (OC) (*n* = 6). H-Score of each sample was analyzed at least nine different areas by color deconvolution of ImageJ software and data are mean ± SD. Two-tailed unpaired Student’s *t* test. ****p < 0.0001. **c** Dual-color immunofluorescence study showed that about 66.3% of LGR5 signal was co-localized with the expression of pan-cytokeratin (Pan-CK) in the solid AM tissues (*n* = 3). About 68.6% and 74.1% of LGR5^+^ cells co-expressed ALDH1 and OCT4, respectively. Scale bars, 20 μm. The representative images are from the follicular AM. **d** The quantification of correlation coefficient of LGR5 and epithelial biomarker (Pan-CK) and stem cell-related genes (ALDH1 and OCT4) in the solid AMs (*n* = 3). Each group was calculated at least three different areas by CellProfiler software and data are mean ± SD.

Initially, we explored whether LGR5 represented a putative marker for epithelial stem-like cells in AM. To this purpose, we examined the expression of LGR5 in a total of fifteen human AM tissues (ten follicular type; two plexiform type; three desmoplastic type) versus corresponding normal adjacent tissues (NATs), and six benign odontogenic cysts (OC). IHC studies showed that the LGR5 expression was consistently higher in different histological variants of solid AM (follicular, plexiform, and desmoplastic AM) as compared with OCs, and the corresponding NATs (Fig. 1a, b; Supplementary Fig. S1b). Of note, the overall H-score of LGR5 expression in AM tumor tissues was about fourfold higher than that in normal control and OCs (Fig. 1b). The immunoreactive signals of LGR5 expression are mainly localized in the AM epithelial islands, with an average 70.45% of LGR5^+^ cells in epithelial islands versus an average 18.62% of LGR5^+^ cells in stroma of the total fifteen AM tissues (Supplementary Fig. S1a). The expression of LGR5 in AM epithelial islands was slightly higher in the plexiform (85.98%) than that in the follicular type (66.50%) (*p* < 0.05). Dual-color immunofluorescence (IF) study showed that about 66.3% of LGR5 signal was co-localized with pan-cytokeratin (Pan-CK) expression in AM tissues, indicating that LGR5 was mainly expressed by epithelial cells in AM (Fig. 1c, d; Supplementary Fig. S2a, b). Since the solid type of AM accounts for about 80% of all AM cases and has a high recurrence rate^1,32^, we focus our studies using this major type of AM.

### Characterization of a subpopulation of LGR5^+^ stem-like epithelial cells in AM

We then determined whether LGR5 expression was associated with stem cell-related genes in solid type of AM tissues. Dual-color IF studies showed that within the epithelial islands of all three subtypes of solid type of AMs, LGR5 co-expressed with ALDH1 and octamer-binding transcription factor 4 (OCT4) (Fig. 1c, d; Supplementary Fig. S2a, b), two well-recognized stem cell-regulatory genes in CSCs^33–36^. Further analysis indicated that about 68.6% of LGR5^+^ epithelial cells co-expressed ALDH1 and 74.1% LGR5^+^ epithelial cells co-expressed OCT4 (Fig. 1d). These results suggest that solid type of AM tissues harbor a subpopulation of LGR5^+^ epithelial stem-like cells.

**Fig. 2:**
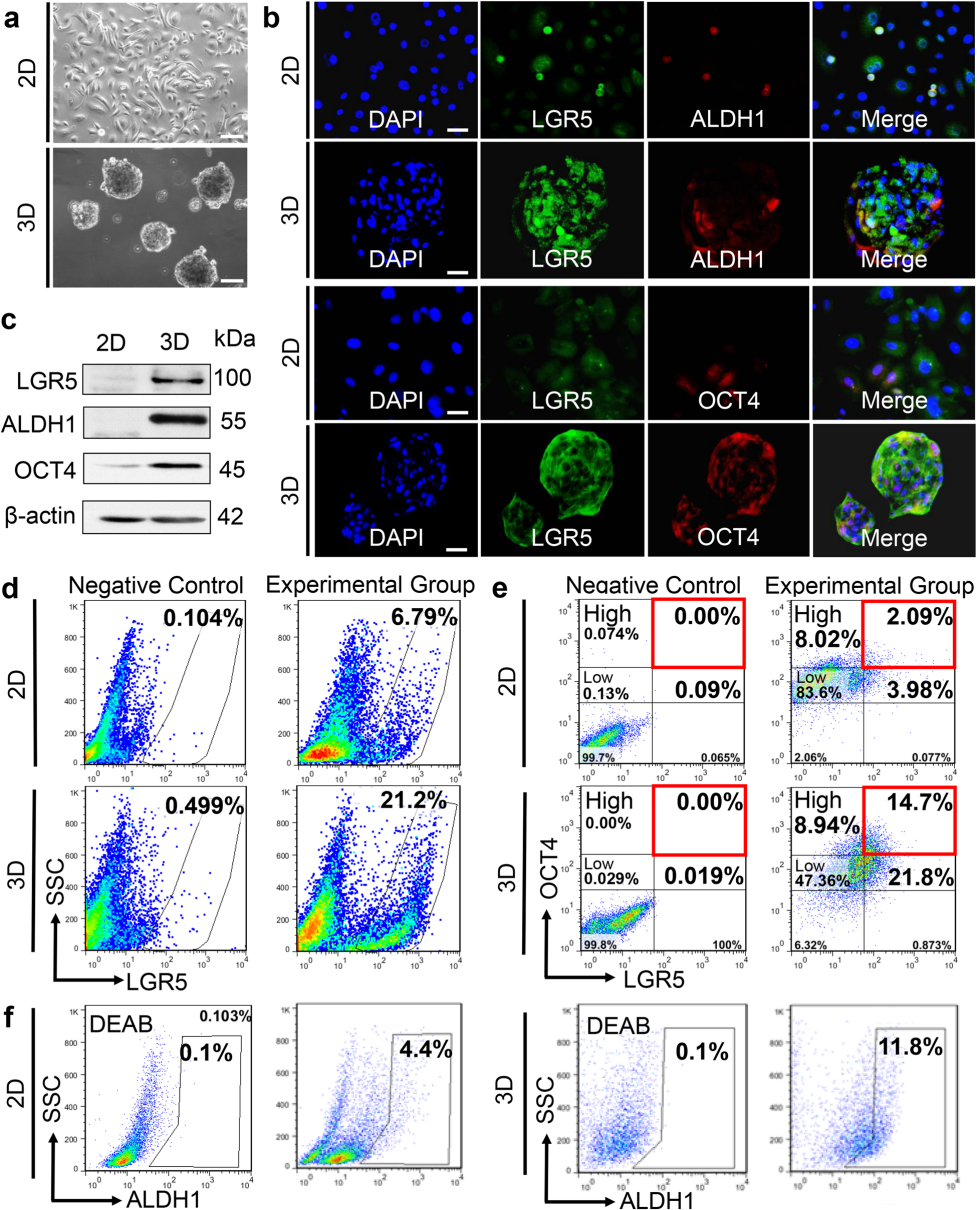
LGR5^+^ALDH1^+^OCT4^High^ AM epithelial cells are enriched in 3D-spheroid culture. **a** Morphology of AM epithelial cells derived from primary human AM tissues and cultured under 2D-monolayer or 3D-spheroid-forming conditions. Scale bars, 100 μm. **b** AM epithelial cells were cultured under 2D-monolayer or 3D-spheroid culture conditions for 5 days. The co-expression of LGR5, ALDH1, and OCT4 was observed by immunofluorescence studies. Scale bars, 20 μm. **c** Augmented expression of LGR5, ALDH1, and OCT4 in AM epithelial cells under 3D-spheroid culture versus 2D-monolayer culture as determined by western blot analysis. **d** About threefold enrichment of LGR5^+^ AM epithelial cells (from 6.79 to 21.2%) under 3D-spheroid culture versus 2D-monolayer culture as determined by flow cytometric analysis. **e** The proportion of LGR5^+^OCT4^Low^, LGR5^+^OCT4^High^, and total OCT4^High^ cells in AM epithelial cells cultured under 2D-monolayer culture and 3D-spheroid conditions was determined by flow cytometry. **f** The activity of ALDH1 was increased by about threefold (4.4 to 11.8%) in AM epithelial cells under 3D-spheroid culture versus 2D-monolayer culture as determined by flow cytometric analysis. All results are representative of at least three independent experiments.

To characterize the stem cell properties of LGR5^+^ epithelial cells in AM, primary epithelial cells derived from solid follicular AM tissues^16^ (Fig. 2a) were cultured under 3D-spheroid forming condition, an approach utilized for self-renewal assay and enrichment of stem cells^37–40^. Our results showed that the expression of LGR5, ALDH1, and OCT4 was significantly increased in AM epithelial cells after 5 days in 3D-spheroid culture as compared with those under 2D culture condition (Fig. 2b–d). Flow cytometric analysis showed that LGR5^+^ cells grown in 3D-spheroid condition were consistently enriched by threefold (from 6–10% to 20–30%) as compared with 2D culture (Fig. 2d). Meanwhile, we further analyzed LGR5^+^OCT4^Low^, LGR5^+^OCT4^High^, and total OCT4^High^ cells in AM epithelial cells. Our results showed that under 3D culture versus 2D culture, both LGR5^+^OCT4^Low^ and LGR5^+^OCT4^High^ cells were significantly enriched, from 3.98% to 21.8% and 2.09% to 14.7%, respectively; and total OCT4^High^ cells were increased from 10.11% (8.02 + 2.09%) to 23.64% (8.94 + 14.7%) (Fig. 2e). Meanwhile, ALDH1 activity increased by about threefold in 3D-spheroid AM epithelial cells over 2D cultures as determined by ALDEFLUOR assay (Fig. 2f). Similarly, LGR5^+^ and LGR5^+^OCT4^High^ cells were enriched while ALDH1 activity increased in immortalized AM-1 cells when cultured under 3D-spheroid culture condition (Supplementary Fig. S3a–c). Collectively, these results suggest that LGR5^+^ALDH1^+^OCT4^High^ AM epithelial cells demonstrate increased self-renewal capability and represent a subpopulation of tumor epithelial stem-like cells in solid AM (AM-EpiSCs).

### LGR5^+^ AM epithelial cells are endowed with intermediate EMT phenotype and stem cell properties in vitro

**Fig. 3:**
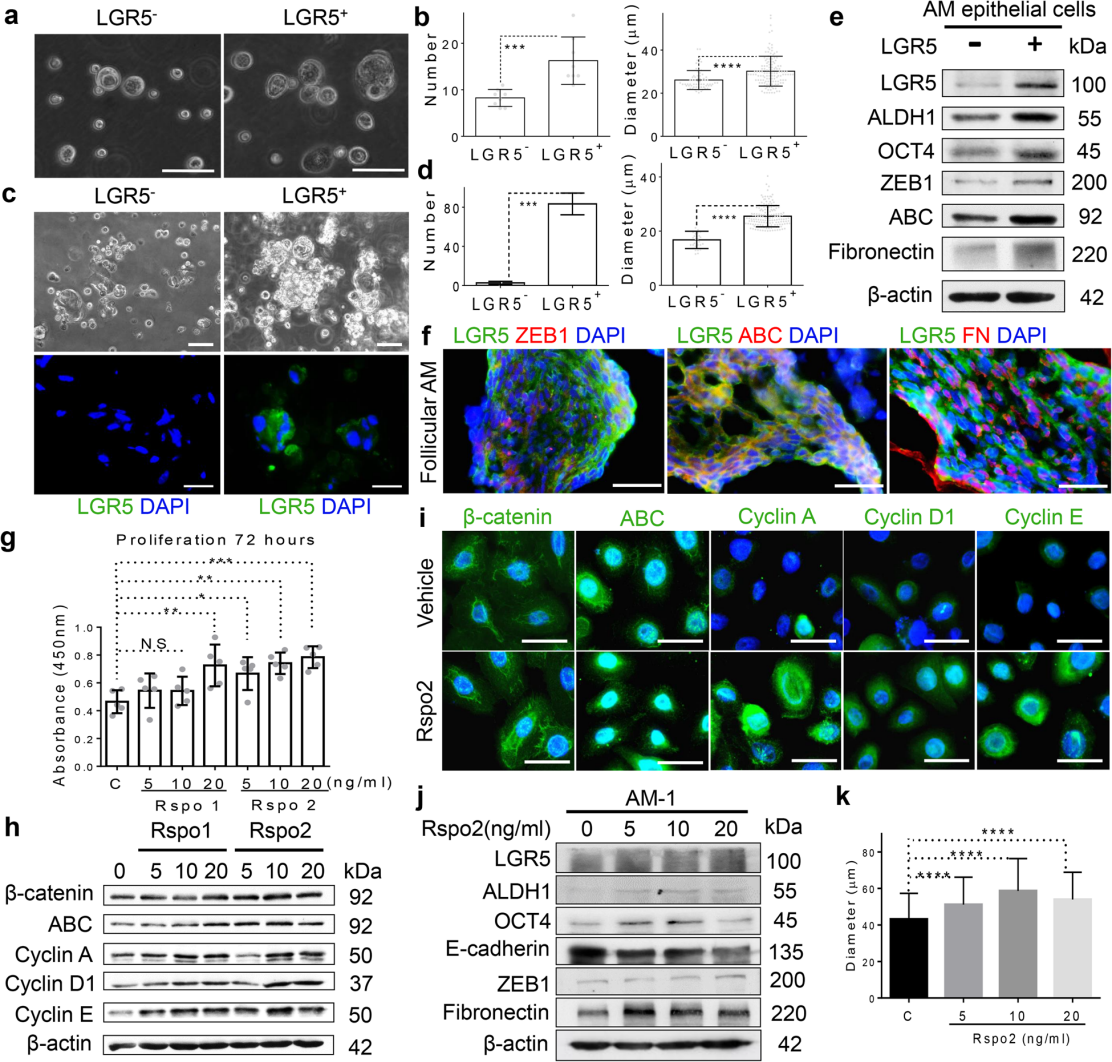
LGR5+ AM epithelial cells exhibit self-renewal ability and EMT phenotypes and are responsive to R-spondin stimulation. **a**, **b** LGR5^+^ AM epithelial cells were sorted out from parental primary AM epithelial cells using LGR5 antibody-conjugated magnetic beads, which showed increased 3D-spheroid-forming ability than their LGR5^−^ counterparts. Scale bars, 50 μm. Data are Mean ± SD (each group was measured eight different random areas under the microscope), two-tailed unpaired Student’s *t* test. ***p < 0.001, ****p < 0.0001. **c** Sorted LGR5^+^ AM epithelial cells formed more and larger 3D-spheroids as compared with LGR5^−^ counterparts after culturing in 3D extracellular matrix (ECM) Matrigel for two weeks. Scale bars, 20 μm. **d** The quantification of spheroid formation as shown in **c**. Data are Mean ± SD (each group was measured three different random areas under the microscope), two-tailed unpaired Student’s *t* test. ***p < 0.001, ****p < 0.0001. **e** Increased expression of stem cell-related genes, ALDH1 and OCT4, and EMT-related genes/markers, ZEB1, active β-catenin (ABC) and fibronectin (FN), in sorted LGR5^+^ AM epithelial cells as compared with that in LGR5^−^ counterparts. **f** Colocalization of LGR5 and specific EMT-related genes/markers, ZEB1, ABC, and FN in the follicular AM tissue. Scale bars, 100 μm **g** Stimulation with R-spondins (Rspo1 and Rspo2) for 72 h increased proliferation in AM epithelial cells in a dose-dependent manner. Mean ± SD, *n* = 5, one-way ANOVA and Dunnett’s post-test for comparing treatments with untreated control. Control = PBS. NS not significant. *p < 0.05, **p < 0.01, ***p < 0.001. **h** Stimulation with R-spondins (Rspo1 and Rspo2) for 48 h increased the expression of active β-catenin (ABC), cyclin A, D1, and E in AM epithelial cells in a dose-dependent manner as determined by western blot analysis. **i** Stimulation with 20 ng/ml of Rspo2 for 48 h increased the expression of active β-catenin (ABC), cyclin A, D1, and E in nuclei of AM epithelial cells as determined by immunofluorescence studies. Scale bars, 20 µm. **j** Stimulation with of Rspo2 for 48 h led to a dose-dependent increase in the expression of ALDH1, OCT4, ZEB1, and fibronectin but decreased the expression of E-cadherin in AM-1 cells as determined by western blot analysis. **k** Stimulation with of 20 ng/ml of Rspo2 for 10 days significantly increased 3D-spheroid formation in AM-1 cells as compared with the control group. Mean ± SD, two-tailed unpaired Student’s *t* test (*n* = 3 in each group). ****p < 0.0001. All results are representative of a*t* least two to three independent experiments.

Next, we characterized the stem cell properties of LGR5^+^ AM epithelial cells. To this end, LGR5^+^ AM epithelial cells were sorted from parental primary epithelial cells using LGR5 antibody-conjugated magnetic beads and confirmed by flow cytometry (Supplementary Fig. S4a). 3D-spheroid forming assay showed that LGR5^+^ AM epithelial cells formed more and larger spheroids than LGR5^−^ counterparts (Fig. 3a, b). Meanwhile, sorted LGR5^+^ AM epithelial cells when cultured in 3D Matrigel for two weeks also readily formed larger 3D-spheroids as compared with LGR5^−^ counterparts (Fig. 3c, d). Similarly, LGR5^+^ cells sorted from AM-1 cells exhibited increased 3D-spheroid forming capability as compared with the parental cells and LGR5^−^ counterparts (Supplementary Fig. S4b, c). These results suggest that LGR5^+^ AM epithelial cells are more capable of self-renewal than their LGR5^−^ counterparts.

Since EMT contributes to cell plasticity and CSC formation^13^, we then compared the expression profiles of stem cell-related and EMT regulatory TFs in sorted LGR5^+^ and LGR5^−^ AM epithelial cells. Western blot analysis demonstrated a robust increase in the expression of ALDH1 and OCT4 as well as EMT-related genes, ZEB1, active *β*-catenin (ABC) and fibronectin, in LGR5^+^ cells sorted from both primary AM epithelial cells and AM-1 cell lines in comparison with their LGR5^−^ counterparts, respectively (Fig. 3e; Supplementary Fig. S4d). In addition, the colocalization of LGR5 and ZEB1, ABC, and fibronectin was further confirmed in different subtypes of solid AM tissues as determined by IF studies (Fig. 3f; Supplementary Fig. S5). Functionally, we found that LGR5^+^ AM epithelial cells exhibited significantly increased migration ability as compared with their LGR5^−^ counterparts (*p* < 0.001) (Supplementary Fig. S4e, f). Taken together, these findings suggest that LGR5^+^ epithelial cells also possess features characteristic of an intermediate EMT phenotype.

### R-spondins stimulates proliferation of AM epithelial cells

We then determined the biological function of LGR5 by stimulating AM epithelial cells with its ligands, R-spondin-1 or -2 (Rspo1 and Rspo2). To this purpose, primary AM epithelial cells were stimulated with Rspo1 and Rspo2 for 48 h, respectively, and the proliferative activity was evaluated. We found that stimulation with either Rspo1 or Rspo2 led to a dose-dependent increase in the proliferation in AM epithelial cells (Fig. 3g). Western blot analysis showed that treatment with Rspo1 and Rspo2 significantly increased the expression of ABC, cyclin A, D1, and E in AM epithelial cells, while the stimulatory effect conferred by Rspo2 was more robust than that by Rspo1 (Fig. 3h). The increased expression of ABC, cyclin A, D1, and E in nuclei of AM epithelial cells following treatment with Rspo2 for 48 h was further confirmed by IF studies (Fig. 3i; Supplementary Fig. S6a). Similarly, stimulation with Rspo2 increased the expression of ABC, cyclin A, D1, and E as well as the percentage of cells at S-phase (from 8.27% to 12.4%) in immortalized AM-1 cells (Supplementary Fig. S6b, c). Of note, Rspo2 upregulated the expression of ALDH1, OCT4, ZEB1, and fibronectin but decreased the expression of E-cadherin in AM-1 cells (Fig. 3j). Meanwhile, AM-1 cells showed significantly enhanced 3D-spheroid forming capacity upon exposure to Rspo2 as compared with the control (Fig. 3k). These findings suggest that functional LGR5/R-spondin may contribute to AM tumor growth through promoting proliferation, EMT, and acquisition of stem cell properties in AM epithelial cells.

### Lg5^+^ AM epithelial cells possess self-renewal and propagating ability in vivo

**Fig. 4:**
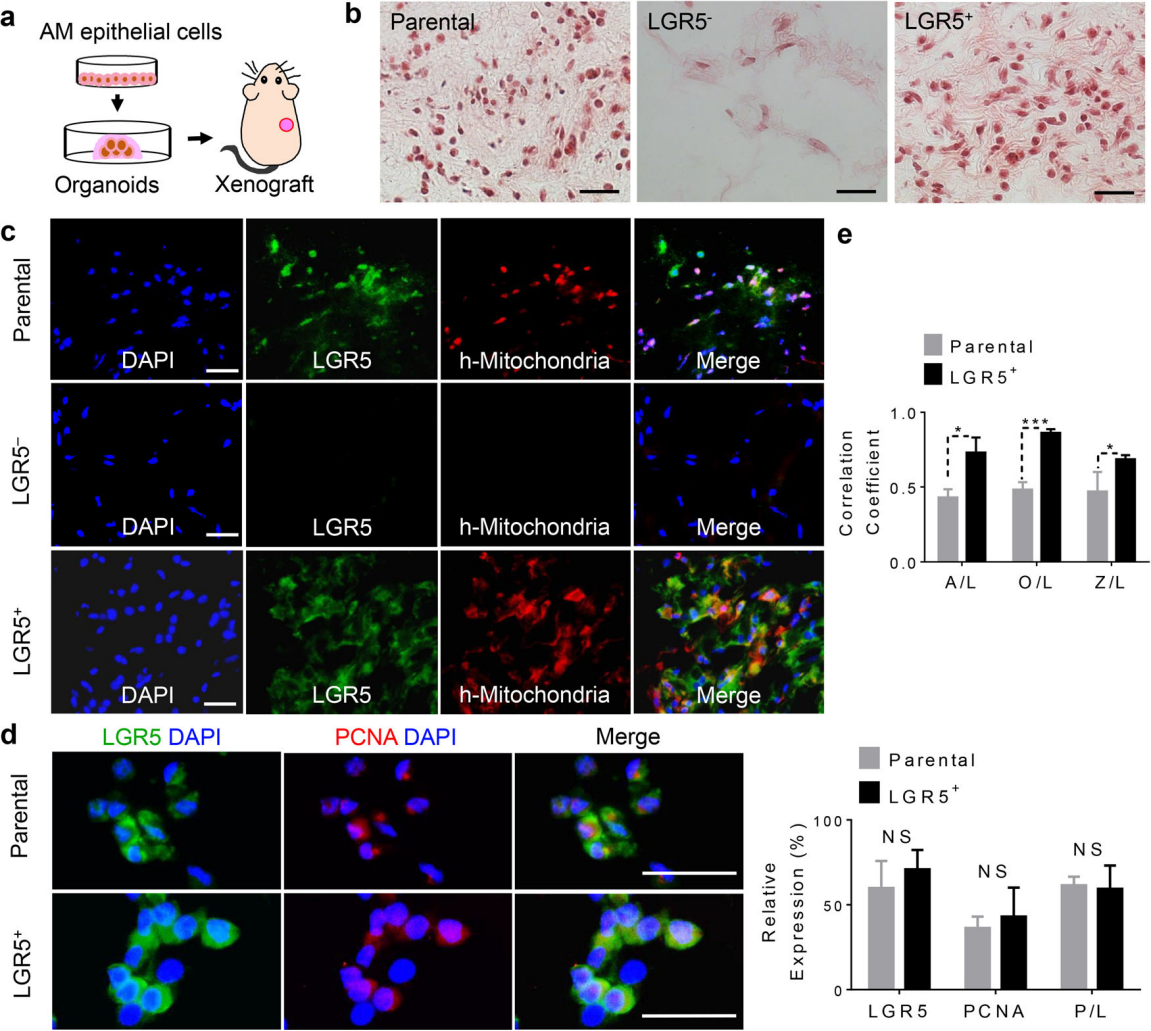
LGR5^+^ AM epithelial cells exhibit propagating ability in vivo. **a** The diagram showing the subcutaneous nude mice model using ex vivo organoids. Following culturing in 3D Matrigel for three weeks, the ex vivo organoids formed by parental, sorted LGR5^+^ or LGR5^−^ AM epithelial cells were harvested and subcutaneously transplanted into the flank of nude mice. **b** Two weeks post transplantation, the tumor-like structures formed in nude mice were harvested for histological analysis by H&E staining. The LGR5^−^ AM epithelial cells were mostly resorbed while the LGR5^+^ and parental groups could generate some tumor-like structure. Scale bars, 20 μm. **c** Immunofluorescence study showed colocalization of human mitochondria and LGR5 in xenografted tumor-like structures formed by transplanted LGR5^+^ and parental AM epithelial cells, but not by LGR^−^ counterparts. Scale bars, 50 µm. **d** Co-expression of LGR5 and proliferating cell nuclear antigen (PCNA) in xenografted tumor-like structures formed by transplanted parental or LGR5^+^ AM epithelial cells as determined by immunofluorescence studies (Left panels). Scale bars, 100 µm. Right panel: the quantification of relative expression of PCNA, LGR5 and PCNA/LGR5 (P/L) from the results shown in the left panels. Mean ± SD (*n* = 4 in each group, xenografts of AM epithelial cells, each group was measured five different random areas under the microscope, two-tailed unpaired Student’s *t* test. NS = not significant). **e** LGR5^+^ AM epithelial cell formed xenografts showed significantly higher co-expression of ALDH1/LGR5 (A/L), OCT4/LGR5 (O/L), and ZEB1/LGR5 (Z/L) than those formed by parental AM epithelial cells. Data are mean ± SD (*n* = 4 in each group, xenografts of AM epithelial cells, each group was measured five different random areas under the microscope, two-tailed unpaired Student’s *t* test. *p < 0.05, ***p < 0.001).

Next, we evaluated the self-renewal capability of LGR5^+^ AM epithelial cells in vivo. To this purpose, parental and sorted LGR5^+^ and LGR5^−^ AM epithelial cells were cultured in Matrigel in vitro for 3 weeks, and then subcutaneously transplanted into the flank of nude mice (Fig. 4a). At day 14 post transplantation, histological analysis showed that parental and LGR5^+^ AM epithelial cells could proliferate and generate tumor-like structures, but transplanted LGR5^−^ cells could not survive and were almost completely resorbed (Fig. 4b), wherein the presence of human AM epithelial cells in vivo was confirmed by immunostaining with a specific antibody for human mitochondria (Fig. 4c). Meanwhile, in the tumor-like structures formed by transplanted parental and LGR5^+^ AM epithelial cells, about 60% of cells co-expressed LGR5 and proliferating cell nuclear antigen (PCNA) (Fig. 4d). Further analysis showed that the percentage of cells co-expressing LGR5 and ALDH1, LGR5 and OCT4, and LGR5 and ZEB1 in tumor-like structures formed by LGR5^+^ AM epithelial cells was significantly higher than that in those formed by parental AM epithelial cells (Fig. 4e; Supplementary Fig. S7). To further evaluate the self-renewal capability of LGR5^+^ AM epithelial cells in vivo, we cultured sorted LGR5^+^ AM epithelial cells in Matrigel for 2 weeks, and then performed cell-dilution assay by subcutaneously transplanting different number of cells (10^6^, 10^5^, 10^4^, and 10^3^) into nude mice. Our results showed that 1-month post transplantation, the implanted LGR5^+^ AM epithelial cells in all groups with decreasing cell numbers survived and formed xenografts with decreasing sizes (Supplementary Fig. S8). Taken together, these results suggest that LGR5^+^ AM epithelial cells possess self-renewal and propagating capability in vivo.

### Generation of ex vivo AM 3D-organoid model with AM epithelial cells

**Fig. 5:**
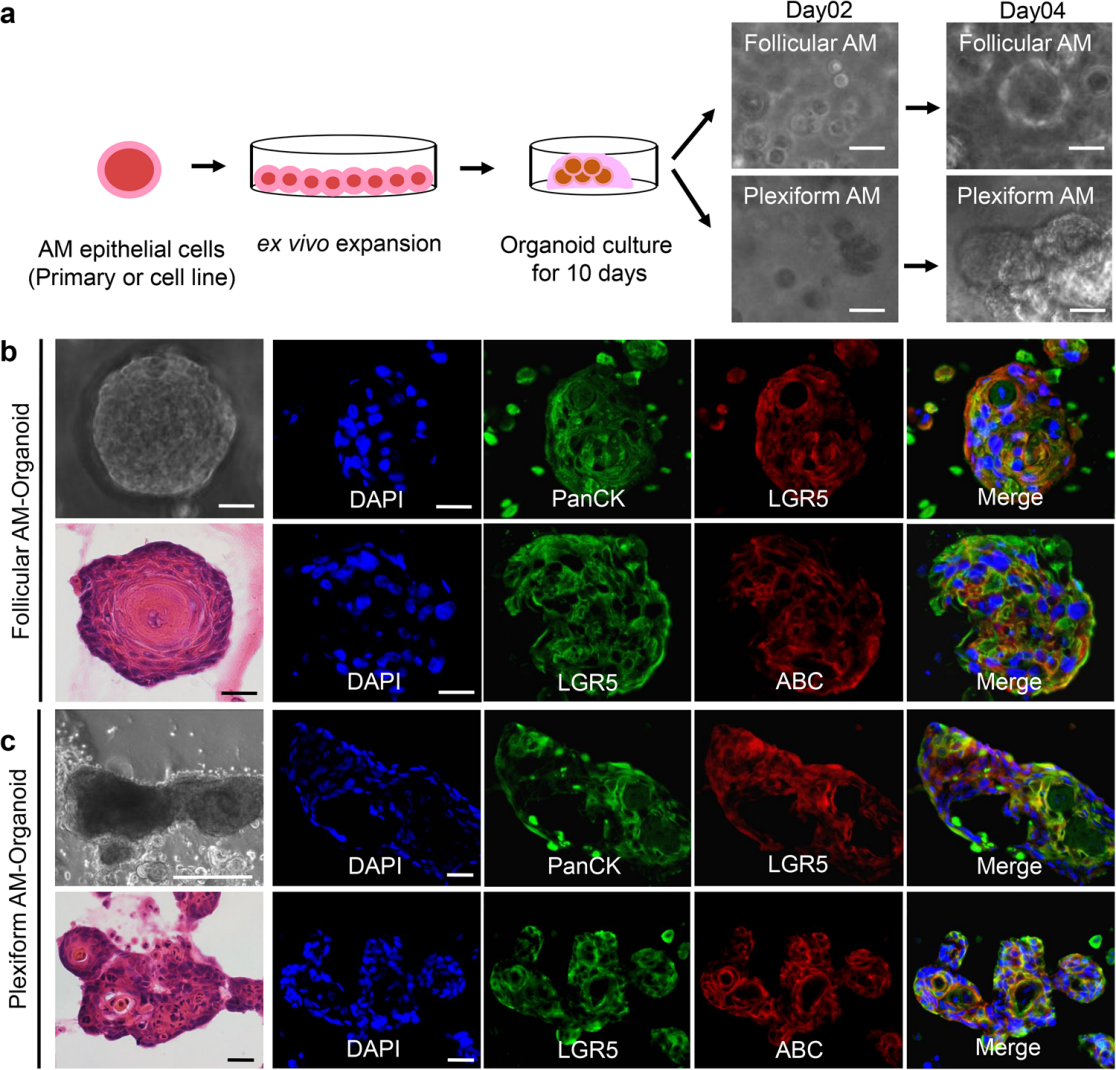
3D organoids recapitulate histopathological features of AM. **a** A diagram showing the generation of 3D organoids by AM epithelial cells. Ex vivo expanded primary AM epithelial cells or AM-1 cells were transferred to 3D Matrigel and cultured for different days. Scale bars, 20 μm. **b** 3D-organoid culture of follicular AM epithelial cells for 10 days. Left, H&E staining showed AM epithelial cells arranged into follicular-type organoids. AM organoids recapitulated certain histopathological features of AM, including hyperchromic nuclei cuboidal (ameloblast-like) peripheral cells arranged in a palisading-like pattern and showed reverse polarity. Right: LGR5 was enriched and co-expressed with pan-cytokeratin and activated *β*-catenin (ABC) in 3D organoids formed by AM epithelial cells as determined by immunofluorescence studies. Scale bars, 20 μm. **c** 3D-organoid culture of AM-1 (plexiform type) for 10 days. Left: H&E staining showed generated organoids with irregular epithelial islands connected as anastomosing strands that were similar to the histopathological features of plexiform AM. Right, LGR5 was enriched and co-expressed with pan-cytokeratin and activated *β*-catenin (ABC) in 3D organoids formed by AM epithelial cells as determined by immunofluorescence studies. Scale bars, left upper: 200 μm; left lower and right: 20 μm. All results are representative of three independent experiments.

Up to date, there is still lack of a preclinical animal model to study benign tumor of jaw bone, especially AM. Here, we explored feasibility to generate the human AM 3D organoids as a preclinical model for this benign/aggressive tumor. To this purpose, we cultured primary AM epithelial cells derived from follicular AM tissues or immortalized AM-1 cells in Matrigel and defined organoid culture medium. We observed formation of 3D-organoid-like structure by both primary AM epithelial cells and AM-1 cells at day 2 following organoid culture (Fig. 5a). At day 10, the organoids were harvested for further analysis. Histologically, the generated AM organoids recapitulated the distinct histopathologic features of follicular and plexiform subtypes of solid AM (Fig. 5b, c). Specifically, organoids generated from primary follicular AM epithelial cells displayed hyperchromic nuclei cuboidal (ameloblast-like) peripheral cells arranged in a palisading-like pattern and demonstrated reverse polarity (Fig. 5b); while organoids generated from AM-1 cells (plexiform type) exhibited irregular epithelial islands connected as anastomosing strands (Fig. 5c). Interestingly, highly co-expression of LGR5 and ABC was also observed in organoids generated from both primary follicular AM epithelial cells and plexiform AM-1 cell line (Fig. 5b, c), similar to that observed in both subtypes of solid AM tissues (Fig. 3f; Supplementary Fig. S5). These findings have demonstrated, for the first time, the feasibility to generate ex vivo human AM 3D organoids, which recapitulated the histopathological features of AM subtypes and further confirmed the potential role of LGR5^+^ EpiSCs in the pathogenesis of AM.

### LGR5^+^ AM-EpiSCs resistant to BRAF^V600E^ inhibitor are capable of tumor formation ex vivo

**Fig. 6:**
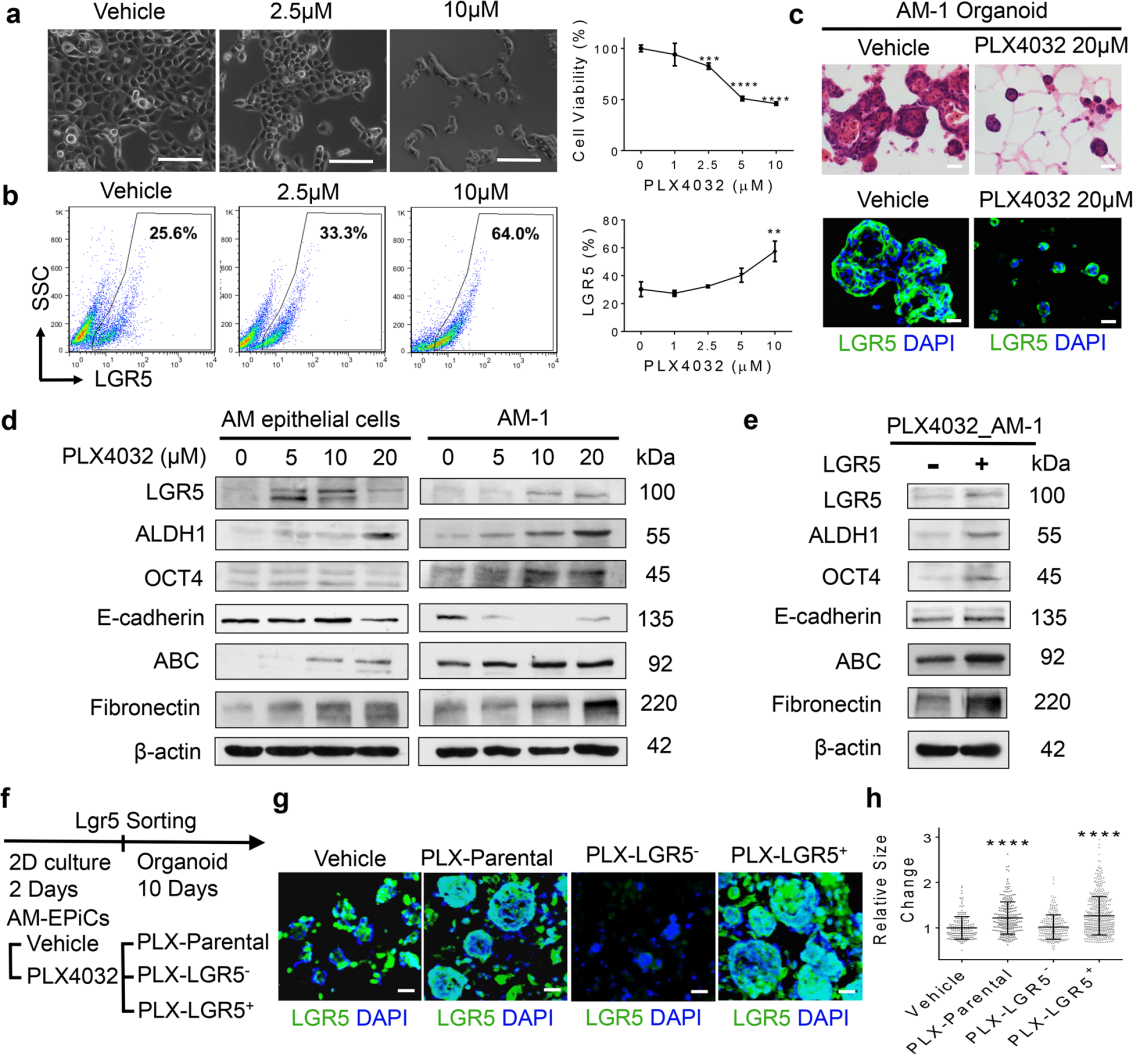
LGR5^+^ AM-EpiSCs resist BRAF^V600E^ inhibitor and drug-resistant LGR5^+^ AM-EpiSCs possess propagating ability to generate AM organoids. **a** Left: AM-1 cells were seeded on a 24-well plate in a cell density of 10^5^/well and treated with different concentrations of a specific BRAF^V600E^ inhibitor (vemurafenib, PLX4032) for 48 h (*n* = 3). Residual cells arranged into irregular epithelial islands connected as anastomosing strands. Right: AM-1 cells were seeded into 96-well plates (5 × 10^4^ cells/well) followed by exposure to different concentrations of PLX4032 for 48 h and the cell viability was determined by cell count kit-8. Data are Mean ± SD, *n* = 4, two-tailed unpaired Student’s *t* test. ***p < 0.001, ****p < 0.0001. **b** Left: enriched expression of LGR5 on AM-1 cells following treatment with different concentrations of PLX4032 for 48 h as determined by flow cytometry. Right: graphs showing the results from flow cytometric analysis as shown in the left panels. Data are Mean ± SD, *n* = 3, two-tailed unpaired Student’s *t* test. **p < 0.01. **c** 3D organoids formed by AM-1 cells for 4 days were treated with PLX4032 (20 μM) for 6 days. Upper: PLX4032 interfered with 3D-organoid formation as determined by H&E staining. Lower: the residual PLX4032-resistant AM-1 cells in 3D organoids were positive for LGR5 as determined by immunofluorescence study. Scale bars, 20 μm. **d** LGR5^+^ epithelial cells were enriched with a concomitant dose-dependent increase in the expression of ALDH1, OCT4, active *β*-catenin (ABC) and fibronectin but decreased E-cadherin expression in either primary AM epithelial cells or AM-1 cells following treatment with different concentrations of PLX4032 under 2D-monolayer culture condition for 48 h. **e** AM-1 cells were treated with PLX4032 (20 μM) under 2D-monolayer culture conditions for 48 h, and then LGR5^−^ and LGR5^+^ AM epithelial cells were sorted by anti-LGR5 microbeads. The expression of stem cell- and EMT-related genes/markers was significantly increased in LGR5^+^ AM epithelial cells in comparison with that in LGR5^−^ counterparts as determined by western blot analysis. **f**, **g** AM-1 were treated with PLX4032 (20 μM) or vehicle under 2D-monolayer culture condition for 48 h and LGR5^−^ and LGR5^+^ AM epithelial cells were sorted by anti-LGR5 microbeads. Unsorted PLX4032-treated parental cells (PLX-parental) and sorted PLX4032-treated LGR5^+^ (PLX-LGR5^+^) cells generated significantly larger and more organoids than the PLX-LGR5^−^ counterparts and even the vehicle-treated parental group. Vehicle: Dimethyl sulfoxide (DMSO). **h** Measurements of organoid size as shown in g Data are Mean ± SD, *n* = 3, one-way ANOVA and Dunnett’s post-test. ****p < 0.0001. All results are representative of at least two to three independent experiments.

Previous studies have shown that about 46–82% of AM cases exhibit BRAF^V600E^ mutation^4–9^. We also confirmed BRAF^V600E^ mutation in solid type of AM tissues by IHC studies (Supplementary Fig. S9a). We then determined whether treatment of AM-1 cells with a specific BRAF^V600E^ inhibitor, vemurafenib (PLX4032), could target the subpopulation of LGR5^+^ cells in AM epithelial cells. Our results showed that treatment with PLX4032 reduced cell viability in a dose-dependent manner (Fig. 6a). Interestingly, flow cytometric analysis revealed that exposure to PLX4032 resulted in a dose-dependent enrichment of LGR5^+^ cells (Fig. 6b). Meanwhile, PLX4032 apparently interfered with organoid formation but most of the viable cells were positive for LGR5 (Fig. 6c; Supplementary Fig. S9b). Western blot analysis confirmed that treatment with PLX4032 not only enriched LGR5 expression but also enhanced the expression of ALDH1, OCT4, ABC, and fibronectin, and decreased the expression of E-cadherin in both primary AM epithelial cells and AM-1 cell lines (Fig. 6d).

We next sorted out LGR5^+^ and LGR5^−^ cells from AM-1 cells following treatment with PLX4032 and compared the expression of these stem cell- and EMT-related genes. The expression of ALDH1, OCT4, ABC, and fibronectin was markedly elevated in sorted PLX4032-resistant LGR5^+^ cells as compared with their LGR5^−^ counterparts (Fig. 6e). In addition, we determined the organoid-forming capacity of PLX4032-resistant LGR5^+^ AM-1 cells. To this end, AM-1 cells were treated with 20 *μ*M of PLX4032 under 2D culture condition for 48 h. Afterward, unsorted parental (PLX-parental), sorted LGR5^+^ (PLX- LGR5^+^) and LGR5^−^ (PLX-LGR5^−^) cells were cultured in organoid culture condition, respectively, while AM-1 cells treated with vehicle (DMSO) were used as control (Fig. 6f). Our results showed that PLX4032-treated AM-1 cells (PLX-parental cells) formed significantly more and larger organoids than AM-1 cells treated with vehicle (Fig. 6g, h). More compellingly, LGR5^+^ cells sorted from PLX4032-treated AM-1 cells (PLX- LGR5^+^) displayed more abundant and larger organoids than both their LGR5^−^ counterparts (PLX-LGR5^−^) and AM-1 cells treated with vehicle (Fig. 6g, h). Taken together, these findings have demonstrated that LGR5^+^ AM-EpiSCs can surmount resistance to the BRAF^V600E^ inhibitor (PLX4032) and these PLX4032-resistant LGR5^+^ AM-EpiSCs are endowed with stem cell properties and an intermediate EMT phenotype with enhanced capacity for tumor organoid formation.

## Discussion

Most solid tumors are composed of heterogeneous populations of tumor cells with subpopulations endowed with increased self-renewal and tumor repropagating capabilities termed CSCs or TICs^11^. To date, a panel of cell surface molecules has been utilized to identify CSCs in distinct types of cancer^11,41^. LGR5, upon binding with R-spondins, triggers the activation of downstream Wnt/*β*-catenin signaling pathway^17^, and has also been used as a putative marker for CSCs in several types of cancers^20–25^. However, like other adult stem cells, no single molecule serves an exclusive marker for a specialized CSC compartment. Multiple markers alone or in combination with ALDH enzyme activity and/or the expression of stemness-regulatory genes, like OCT4, Nanog, and SOX2, have been utilized to identify a special CSC subpopulation in different tumors^41^. Even though the critical role of CSCs or TICs in tumorigenesis, progression, and therapeutic relapse has been extensively explored and CSC-targeting therapies are emerging as novel strategies in therapeutics of various cancers^11,12^, limited work has been done to explore the potential role of tumor stem cells in the pathogenesis and therapy of various benign tumors of jaw bones, e.g., AM. Developmentally, AM may have possibly derived from remnants of odontogenic epithelium, the migrating epithelium at the cervical loop, and lining of odontogenic cyst^1,27^, while LGR5 has been well recognized as putative marker for odontogenic epithelial stem cells^28–31^. Herein, we demonstrated that different subtypes of solid AM tissues harbor a subpopulation of LGR5^+^ epithelial cells co-expressing stemness-related genes such as ABC, OCT4, and ALDH1 as well as EMT-related genes such as ZEB1 and fibronectin, all of which were significantly increased in isolated epithelial cells when cultured under 3D-spheroid-forming conditions (Figs. 1–3; Supplementary Figs. S2 and S3). Meanwhile, purified LGR5^+^ epithelial cells displayed enhanced capacities to form 3D-spheroid in vitro and to generate tumor-like structures in vivo (Figs. 3 and 4). These findings support the notion that LGR5^+^ epithelial cells in AM represent a subpopulation of epithelial tumor stem-like cells (LGR5^+^ AM-EpiSCs) harboring an intermediate EMT phenotype, which may contribute to the pathogenesis and recurrence of this benign/yet aggressive odontogenic tumor.

Accumulating evidence support the notion that CSCs represent a dynamic or plastic status, whereby tumor cells can convert or reprogram between stem and nonstem cell state due to the signals they encounter within the tumor microenvironment (TME), e.g., chronic inflammation and therapeutic insults^11,13^. The dynamic bidirectional phenotypic conversion between non-CSCs and CSCs may contribute to the development of heterogeneity of CSCs, e.g. distinct quiescence, therapeutic sensitivity, and capabilities for EMT, invasion, and metastasis^11,13^. EMT is a complex reprogramming process through which epithelial cells acquire a mesenchymal or epithelial/mesenchymal hybrid cell phenotype, which plays an important role in regulating plasticity of CSCs^13–15^. A large panel of growth factors, cytokines, chemokines and other stimuli within the TME can trigger epithelial tumor cells to undergo EMT and acquire stem cell properties^41^. In the present study, we showed that Rspo2 could stimulate the proliferation and induce EMT in AM epithelial cells, suggesting that LGR5/Rspo2 may functionally contribute to the development and maintenance of the hybrid EMT phenotype of AM-EpiSCs.

Traditional 2D-monolayer cell culture has long been a mainstay in the field of biomedical research. However, it is a great challenge to maintain the intrinsic cell properties and retain the molecular and epigenetic repertoire due to the lack of supporting niche factors^42,43^. In recent years, 3D-organoid culture is emerging as a novel approach in cell biology, particularly in stem cell research and cancer cell biology, which enables modeling the TME and maintaining the major genetic and phenotypic features of individual tumors in an efficient and cost-effective manner^43–45^. Organoid and spheroid cultures allow for better modeling cell behaviors in a recapitulating in vivo-like natural TME such as cell–cell interactions, hypoxia, pH gradients, extracellular matrix (ECM), and different profile of bioactive molecules^46^. For instance, the cell–cell and cell–ECM interactions and cell geometry in 3D culture can increase self-renewal ability and the expression of stem cell-related genes, such as OCT4 and Nanog^47–49^, thus allowing for maintenance and expansion of normal and CSCs^46^. Under most conditions, 3D-organoid cultures require mouse-derived ECM substitutes with variant stiffness or rigidity e.g. Matrigel or basement membrane extract, which may affect the outcome of experiments^43^. Most recently, mechanically and chemically defined hydrogel matrices with controllable substrate stiffness and rigidity have been developed for organoid culture of patient-derived colorectal tumors^50^. In the present study, we utilized mouse-derived ECM for AM-organoid culture, which led to maintenance and expansion of LGR5^+^ AM-EpiSCs (Figs. 3 and 5). However, further studies are warranted to explore the mechanisms whereby mechanical properties of ECM and other factors enhance the self-renewal and expansion of this subpopulation of AM-EpiSCs. In addition, organoids can be used, to certain degrees, as preclinical alternative models to animal models because they can reduce experimental complexity, allow real-time imaging and high-throughput screening, and enable the study of diseases that are not easily and accurately modeled in animals^51^. To date, numerous organoids have been reported for various types of malignant cancers for multiple purposes^43–45^. However, much less work has been done to develop an organoid platform to model a benign tumor. In this study, we have demonstrated for the first time the feasibility to generate 3D-organoid structures of AM by using AM epithelial cells, which recapitulated the histopathological features and LGR5 expression profile of distinct subtypes of solid AM (Fig. 5). However, it is noteworthy that, even though a short-term subcutaneous transplantation of AM 3D organoids into nude mice led to xenograft formation (Fig. 4), demonstrating the self-renewal and propagating capabilities of LGR5^+^ EpiSCs in vivo, we still cannot establish an appropriate animal model for long-term observation of the role of this unique subpopulation of epithelial stem-like cells in the initiation and progression of AM, a common challenge for most types of benign tumors due to their intrinsically benign and slow progression properties.

BRAF^V600E^ mutation has been implicated in the progression of several types of carcinoma by RAS-independent activation of MEK/ERK signaling pathways^52^, which has also been reported in about 46–82% of AM cases^4–9^. To date, several small molecule inhibitors that specifically target BRAF^V600E^ mutation have been developed as potential therapeutic drugs for tumors with this mutation, including an active clinical trial (NCT02367859) with the combinatory use of dabrafenib and trametinib in the treatment of AM, but the development of intrinsic and acquired resistance has significantly hindered their application^52^, but the underlying mechanisms are still elusive. In the present study, we utilized the 3D-organoid platform generated by AM epithelial cells to evaluate their responses to treatment with vemurafenib (PLX4032), a selective inhibitor of BRAF^V600E^ mutation. Even though PLX4032 interfered with cell viability and organoid formation by AM epithelial cells, it simultaneously enriched the proportion of LGR5^+^ AM-EpiSCs with EMT phenotype and enhanced capacity for organoid formation (Fig. 6), suggesting that LGR5^+^ AM-EpiSCs are resistant to a selective BRAF^V600E^ inhibitor.

In conclusion, this is the first study to identify a subpopulation of LGR5^+^ epithelial cells endowed with tumor stem-like cell properties and intermediate EMT phenotype in solid AM (LGR5^+^ AM-EpiSCs), which may play an important role in its pathogenesis and recurrence. In addition, we established conditions for generation of 3D AM organoids which recapitulate certain degree of different histological subtypes of AM, thus allowing us to generate 3D AM organoids by directly using both biopsy and final excisional tissues from AM in the future. In the short term, the human AM 3D organoids may be utilized as a platform for further mechanistic studies and screening small molecules that can specifically target LGR5^+^ AM-EpiSCs due to the lack of an animal model for AM. In the long run, further studies are warranted to optimize the conditions for generation and transplantation of 3D AM organoids in order to generate a consistent animal model of AM for deep mechanistic and interventional studies in vivo.

## Materials and methods

### Tissue collection

The study was conducted in accordance with human subject research guidelines and a protocol approved by the Institutional Review Board (IRB) at University of Pennsylvania (UPenn) (IRB#817407) and focused on solid AM, the most common histopathological variant of this benign tumor with a high recurrent rate. Three fresh primary solid AM samples, including one follicular type, one follicular/plexiform mixed type, and one desmoplastic type, were obtained immediately post-surgical procedures from the Department of Oral and Maxillofacial Surgery of Penn Medicine Hospital of UPenn. Meanwhile, we also collected six dentigerous cysts as the control. In addition, a total of 12 formalin-fixed paraffin-embedded blocks of solid AM samples were retrieved from the archives at Departments of Pathology of UPenn School of Dental Medicine (IRB#817407), Dongguan Hospital Affiliated to Medical College of Jinan University, and the Fifth People’s Hospital of Dongguan, which were also approved by the research and ethical committee of the two hospitals in China (Guangdong, China). Informed consent was obtained from all subjects. Diagnoses were made by two independent pathologists, including a board-certified oral and maxillofacial pathologist, based on the WHO classification (2017) of odontogenic tumors.

### Cell culture

An immortalized AM cell line (AM-1) was generously provided by Dr Hidemitsu Harada at Iwate Medical University and cultured with defined serum-free keratinocyte growth medium (KGM-2 BulletKit, Lonza). Primary AM epithelial cells were isolated as previously described by our group^16^. Briefly, at least 3–6 mm^3^ of fresh human AM tissues were minced into 0.5–1 mm^3^ pieces followed by enzymatic digestion with 0.2% collagenase I (Gibco) for 1 h in a 37 °C shaking incubator. The dissociated cells were seeded in gelatin-coated tissue culture dishes (2 × 10^4^/cm^3^) in defined KGM-2 culture medium (Lonza) at 37 °C in a humidified incubator with 5% CO_2_. After 48 h, the nonadherent cells were removed, and fresh media were replenished every three days. When cells were at 75–95% confluence, they were subcultured following cell dissociation with 1× Accutase solution (Sigma). The ex vivo expanded epithelial cells were characterized by immunocytochemical and flow cytometric analyses on the expression of epithelial markers, such as E-cadherin and Pan-CK, but negative for MSC markers, such as CD90 and CD105. Early passages of primary cells were cryopreserved, and less than six passages were used for further experiments. Previous studies have shown the difference between primary cells and cell lines, and cell lines may undergo chromosomal rearrangements/duplications or mutations, and epigenetic changes that make cell lines could not recapitulate the primary tumor behaviors^53,54^. Therefore, we performed most experiments by using primary AM cells.

### Immunohistological studies

The human tumor samples were fixed in 4% paraformaldehyde (Santa Cruz) for overnight at 4 °C and embedded in either paraffin or optimal cutting temperature (OCT) compound. For IHC staining, paraffin-embedded sections were deparaffinized, unmasked with Antigen Unmasking Solution, Citric Acid Based (Vector) for 20 min at 95 °C and followed by the protocol of the avidin–biotin complex kit (R.T.U. Universal VECTASTAIN® Elite HRP Kit, Vector). Briefly, sections were incubated at 4 °C overnight with primary antibodies for human LGR5 (Invitrogen, PA5-35304) or BRAF V600E (Invitrogen, MA5-24661). Next day, the VECTASTAIN biotinated universal secondary antibody and reagents were subsequently applied to the sections, followed by color development using the VECTOR NovaRED Peroxidase (horseradish peroxidase, HRP) Substrate Kit (Vector) and counterstained with hematoxylin. Images were observed and photographed under a microscope (Olympus, IX73). Quantification of H-Score was analyzed by Color Deconvolution of ImageJ software^55^.

For dual-color IF study, frozen sections were permeabilized in 0.5% triton X-100 in PBS for 15 min, and then blocked in 2.5% goat serum in PBS at room temperature for 1 h, followed by incubation at 4 °C overnight with a primary antibody for LGR5 (ORIGENE, TA503316, mouse IgG; or Invitrogen, PA5-35304, rabbit IgG) in combination with another primary antibody derived from a different host species, including Pan-CK (BioLegend, 914204), ALDH1 (BD Biosciences, 611194), OCT4 (Abcam, ab18976), ZEB1 (Santa Cruz, sc-25388), non-phospho (Active) *β*-catenin (Cell Signaling, 8814S), fibronectin (Sigma, F3648), human mitochondria (Novus, 113-1), and PCNA (Santa Cruz, sc-7907). Afterwards, the sections were incubated at room temperature for 1 h with appropriate fluorochrome-conjugated secondary antibodies: DyLight™ 488 Donkey anti-rabbit IgG, Alexa Fluor 594 Donkey anti-rabbit IgG, DyLight™ 488 Goat anti-mouse IgG, and Alexa Fluor 594 Goat anti-mouse IgG (BioLegend). Isotype-matched control antibodies (BioLegend) were used as negative controls. Nuclei were counterstained with 4,6-diamidino-2-phenylindole (DAPI) Staining Solution (Abcam) and images were captured with Olympus inverted fluorescence microscope (IX73). Correlation coefficient of dual-color IF study was calculated by CellProfiler software^56^.

### Immunocytochemical studies

Cultured cells in eight-well chamber slides (Millicell EZ SLIDES) were fixed with cold methanol for 15 min at −20 °C. Then cells were incubated with the following primary antibodies at 4 °C overnight: *β*-catenin (Cell Signaling, 8480S), ABC (Cell Signaling, 8814S), cyclin A (Sigma, C4710), cyclin B (Sigma, C8831), cyclin D1 (Cell Signaling, 2926), and cyclin E (Cell Signaling, 4129). The cells were then incubated with appropriate fluorochrome-conjugated secondary antibodies as described above. Isotype-matched control antibodies were used as negative controls. Nuclei were counterstained with DAPI Staining Solution, and then images were captured using Olympus inverted fluorescence microscope (IX73). For quantitative analysis of mean fluorescence intensity, cells with positive signals in at least six random fields were measured by Olympus cellSens software.

### Flow cytometry

AM epithelial cells were harvested and suspended in cell staining buffer (0.5% BSA in PBS with 2 mM EDTA) followed by incubation with primary antibody for LGR5 at 4 °C for 30 min. After washing with PBS, the cells were incubated with appropriate fluorochrome-conjugated secondary antibody in the dark at 4 °C for 30 min. True-Nuclear™ TF Buffer Set (BioLegend) was utilized for OCT4 (Abcam, ab18976) after the membrane staining of LGR5. Isotype-matched IgG control antibodies were used as negative controls. ALDH activity was identified by a nonimmunological method (ALDEFLUOR Kit, STEMCELL) and the inhibitor of ALDH enzyme (ALDEFLUOR DEAB Reagent, STEMCELL) was used as negative controls. Samples were analyzed by BD LSRII flow cytometer. Data were processed and analyzed by FlowJo software.

### Cell proliferation assay

AM epithelial cells were seeded into 96-well culture plates in a density of 1 × 10^4^ cells/well in 100 µl of defined KGM-2 medium with five independent replicates per treatment condition. After 24 h, the cells were washed once with PBS and starved in Keratinocyte basal Medium 2 (KBM2, Lonza) overnight. Then Rspo1 (PeproTech) and Rspo2 (PeproTech) were administrated to the starved AM epithelial cells at concentrations of 0, 5, 10, and 20 ng/ml, respectively. After 72 h, 10 µl of CCK-8 reagent (Cell Counting Kit-8 assay, BioLegend) was added into each well and incubated at 37 °C for 2 h. For drug resistance testing, AM epithelial cells were seeded into 96-well plates (5 × 10^4^ cells/well) and treated with PLX4032 (Cayman Chemical Company) for 48 h and the cell viability was determined by Cell Count Kit-8. The absorbance at 450 nm wavelength was detected using an OPSYS Mr microplate reader (Thermo Fisher).

### Cell cycle analysis

AM epithelial cells were seeded into 35-mm culture dishes at a density of 3 × 10^5^ cells per dish containing 2 ml of defined KGM-2 medium. After 24 h, the cells were washed once with PBS and starved in basal KBM2 medium overnight. Then 20 ng/ml Rspo2 were administrated to the starved AM epithelial cells while nontreated cells were used as the control. Both control and Rspo2-stimulated cells were labeled with Bromodeoxyuridine (BrdU) Labeling Reagent (Invitrogen) overnight, and then harvested after stimulation with Rspo2 for 48 h. Cells were then fixed with 70% cold ethanol for 2 h and permeabilized in 2 N HCl/0.5% Triton X-100 at room temperature for 30 min. Then, the cell pallet was treated with 0.1 M sodium tetraborate (pH8.5) for 2 min followed by washing twice with PBS. Afterward, cells were incubated with a specific mouse monoclonal IgG for BrdU (Sigma, B8434) at room temperature for 1 h followed by incubation with DyLight™ 488 Goat anti-mouse IgG at room temperature for 30 min. After washed cells with PBS, the pellet was resuspended in 0.5 ml PBS containing 10 μg/ml RNase A and 20 μg/ml propidium iodide solution and incubated at room temperature for 30 min in the dark. The samples were analyzed by BD LSRII flow cytometer immediately. Data were processed and analyzed by FlowJo software.

### Western blot

Cell lysates were prepared by incubation with radioimmunoprecipitation assay buffer (Santa Cruz) supplemented with a cocktail of protease inhibitors (Santa Cruz) and the total protein concentrations were determined using bicinchoninic acid method (BioVision). Then 30 µg of proteins were subjected to SDS-polyacrylamide gel electrophoresis before being electroblotted onto a 0.2 μm nitrocellulose membrane (GE Healthcare). After blocking with 5% nonfat dry milk in TBST [25 mmol/l Tris (pH, 7.4), 137 mmol/l NaCl, 0.5% Tween20], membranes were incubated at 4 °C overnight with following primary antibodies: LGR5 (Invitrogen, PA5-35304), ALDH1 (BD Biosciences, 611194), OCT4 (Abcam, ab18976), *β*-catenin (Cell Signaling, 8480S), ABC (Cell Signaling, 8814S), cyclin A (Sigma, C4710), cyclin B (Sigma, C8831), cyclin D1 (Cell Signaling, 2926) and cyclin E (Cell Signaling, 4129), ZEB1 (Santa Cruz, sc-25388), fibronectin (Sigma, F3648), and E-Cadherin (BD Biosciences, 562869). β-actin (Santa Cruz, sc-47778) was used as loading control. After extensively washing, membranes were incubated with HRP-conjugated secondary antibodies (Santa Cruz) and blot signals were developed with ECL^TM^ Western Blotting Detect Reagents (GE Health Care).

### Cell sorting

LGR5^+^ AM epithelial cells were sorted by using magnetic Anti-LGR5 MicroBeads (Miltenyi Biotec) according to the manufacturer’s protocol. Briefly, cultured AM epithelial cells were labeled with Anti-LGR5 MicroBeads at 4 °C for 15 min. After washing, the cell suspension was applied to a LS Colum and separated with a magnetic MACS Manual Separator (Miltenyi Biotec). The purity of sorted LGR5^−^ and LGR5^+^ AM epithelial cells was examined by flow cytometry and confirmed by Western blot with a LGR5 antibody (Invitrogen, PA5-35304).

### Spheroid formation assays

3D-spheroid formation assay was performed as described previously^57–59^. Briefly, unsorted (parental), sorted LGR5^−^ and LGR5^+^ AM epithelial cells were seeded at a density of 5 × 10^4^ cells/well into Ultralow attached six-well plates (Corning) with defined serum-free KGM-2 medium. For 3D-spheroid culture in Matrigel, parental, 5 × 10^5^ of sorted LGR5^−^ and LGR5^+^ AM epithelial cells were suspended in 10 μl KGM-2 medium, mixed with 40 μl Matrigel (Corning), and seeded in 24-well plates with defined serum-free KGM-2 medium. After culturing for 2 weeks, spheroids with a size > 20 μm were counted under Olympus microscope (IX73), and the size and number of spheroids were measured with Olympus cellSens software. To prepare the spheroids in Matrigel for IF study, the whole Matrigel containing spheroids was fixed in 4% PFA for 15 min followed by washing twice with PBS for 15 min each time. The spheroids with Matrigel were detached from the dish by a fine flat spatula and transferred to the mold. The whole Matrigel contained spheroids were embedded in OCT and frozen sections at 10 µm were cut for IF study.

### Migration assay

The migration assay was performed by using 8 μm permeable cell culture inserts in 24-well plate (CELLTREAT 230633) according to the manufacturer’s protocol. The parental AM-1 cells were starved in KBM2 overnight, and then sorted by magnetic Anti-LGR5 MicroBeads. The sorted LGR5 negative and positive AM-1 cells were seeded into the upper chambers of transwells (7 × 10^4^ cells/well) with 200 μl basal KBM2 medium and the lower chambers were filled with 600 μl defined KGM-2 culture medium (*n* = 3 for each group). After 16 h, the transwells were gently washed with PBS twice and nonmigrated cells were removed with cotton rods. Then the migrated cells on transwells were fixed with 70% ethanol for 10 min and dried for 10–15 min. The migrated cells were stained by 0.5% crystal violet in room temperature for 10 min, and then gently washed with PBS. After air dry overnight, the migrated cells were photographed and counted under the microscope.

### 3D-organoid culture

Single-cell suspensions of AM epithelial cells were directly dispersed into growth factor reduced Matrigel (Corning Life Sciences) at a density of 2 × 10^4^ cells/μl (∼1 × 10^6^ each group) and seeded in a drop shape. The dish was inverted during solidification of Matrigel to prevent the cells attaching to the culture dish. After solidified for 20 min, the mixture of the cells and Matrigel were cultured in AM-organoid culture medium: 50% KGM-2 and 50% Dulbecco’s Modified Eagle Medium/Nutrient Mixture F-12 (DMEM/F-12, Thermo Fisher). The organoid formation was observed under a microscope every 2–3 days and the whole Matrigel containing organoids was harvested on day 10. Both H&E staining and IF studies on the expression of Pan-CK, LGR5 and ABC were performed.

### Transplantation of 3D organoids

Eight-week-old female and male athymic NU/J mice were purchased from Jackson Laboratory. All animal procedures were handled according to the guidelines of the Institutional Animal Care and Use Committee of IACUC at UPenn. Mice were group-housed in polycarbonate cages (five animals per cage) in the animal facility with controlled temperature, 40–65% of humidity and a 12-h light/dark cycle. Mice were acclimatized for at least 1 week before the study, fed with a standard laboratory diet and allowed ad libitum access to drinking water. For subcutaneous transplantation, nude mice were randomly assigned into three groups transplanted with 8 × 10^5^ of parental, sorted LGR5^−^ and LGR5^+^ epithelial cells, respectively. Cells were pre-cultured in Matrigel for 2 weeks, and then subcutaneously implanted into the dorsal skin of nude mice (*n* = 4 in each group). Two to four weeks after transplantation, xenografted tumors were harvested for histologic analysis and IF studies on the expression of LGR5, human mitochondria, PCNA, EMT-, and stem cell-related genes. No blinding was carried out for animal experiments.

For the cell-dilution assay, we sorted LGR5^+^ cells from parental primary AM epithelial cells and AM-1 cells, and then cultured in Matrigel (50 μl) for two weeks with different cell numbers: 10^3^, 10^4^, 10^5^, and 10^6^ (*n* = 2 in each group). After 2 weeks, the organoids in Matrigel were transplanted subcutaneously into the dorsal skin of nude mice. Four weeks post transplantation, the volume of transplanted organoid xenografts was calculated; the histology was examined by H&E staining, and the expression of human LGR5 and PCNA was determined by IF studies.

### Statistical analysis

All data are presented as mean ± SD and analyzed using unpaired Student’s *t* test for comparing two groups when appropriate. In cases of multiple groups, statistical analysis was performed through one-way ANOVA analysis with Tukey post-test. All analyses were done using GraphPad Prism. A value of *P* < 0.05 was considered statistically significant.

## Supplementary information

Supplemental Fig 1

Supplemental Fig 2

Supplemental Fig 3

Supplemental Fig 4

Supplemental Fig 5

Supplemental Fig 6

Supplemental Fig 7

Supplemental Fig 8

Supplemental Fig 9

Supplemental figure legends
