# Supplementary figures and images for: LGR5+ epithelial tumor stem-like cells generate a 3D-organoid model for ameloblastoma
Source: Cell Death Dis. 2020 May 7;11(5):338. doi: 10.1038/s41419-020-2560-7 (PMC7206107; doi:10.1038/s41419-020-2560-7)

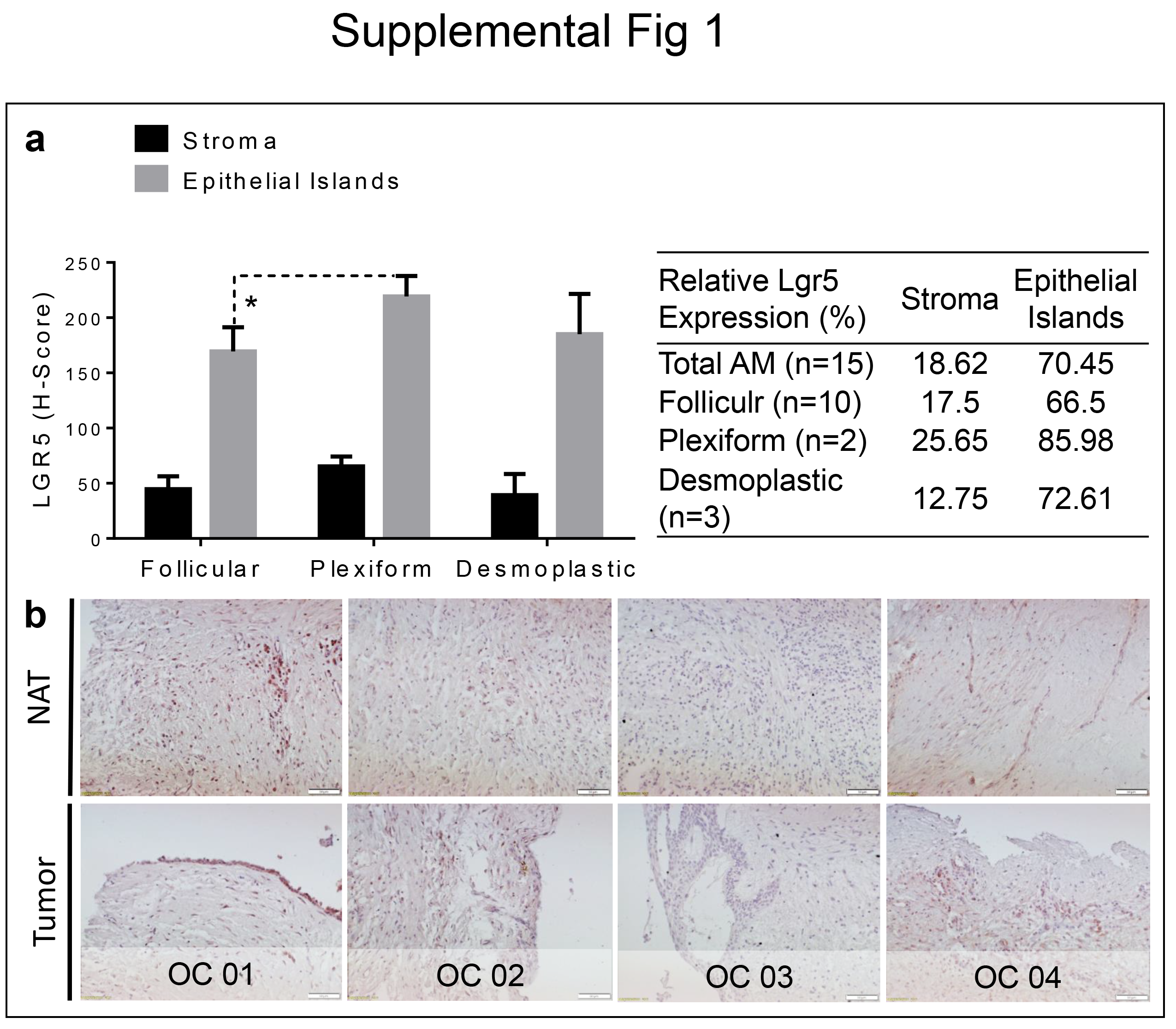

Supplement: Supplementary file 1 — Supplemental Fig 1 [file 41419_2020_2560_MOESM1_ESM.png]

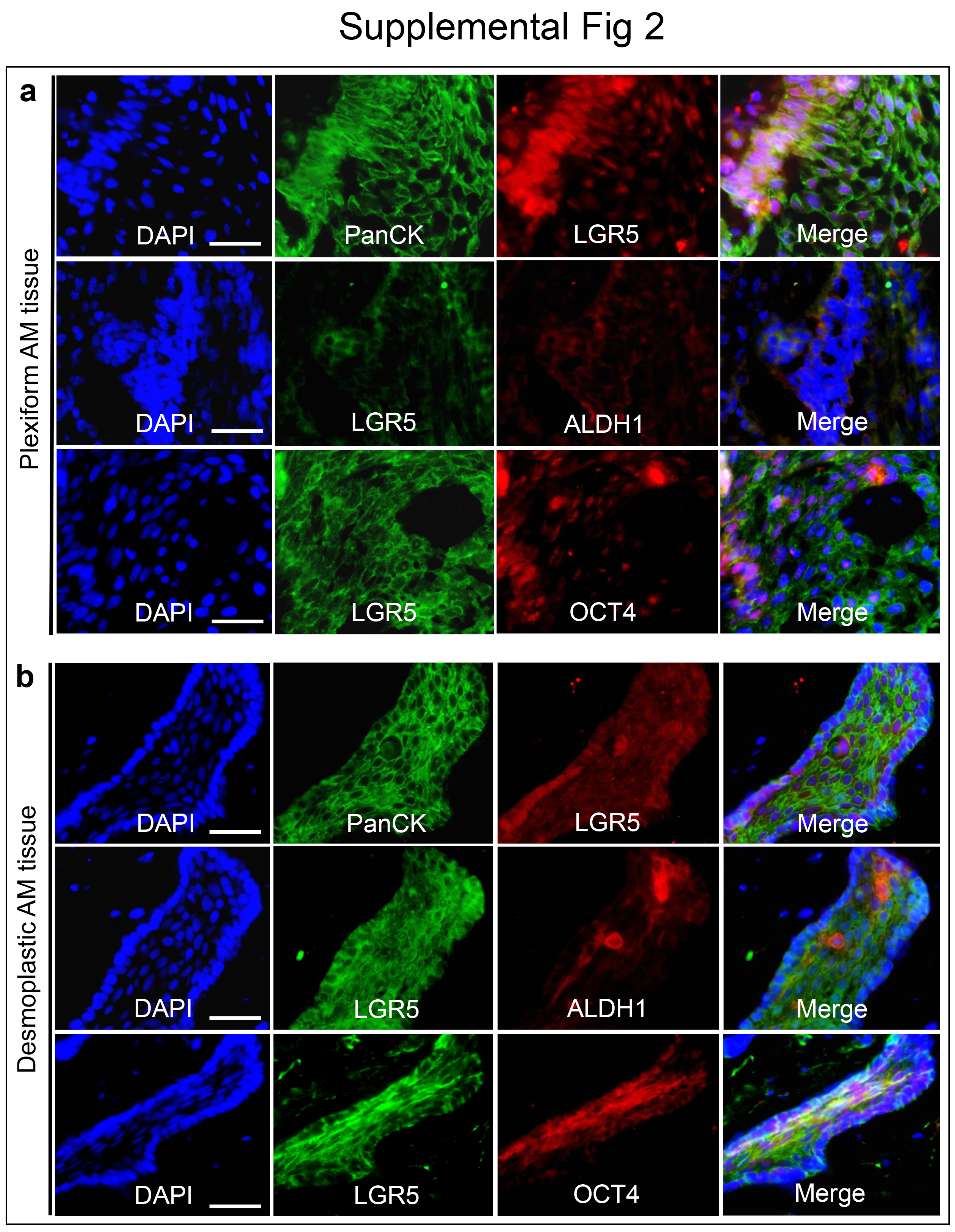

Supplement: Supplementary file 2 — Supplemental Fig 2 [file 41419_2020_2560_MOESM2_ESM.png]

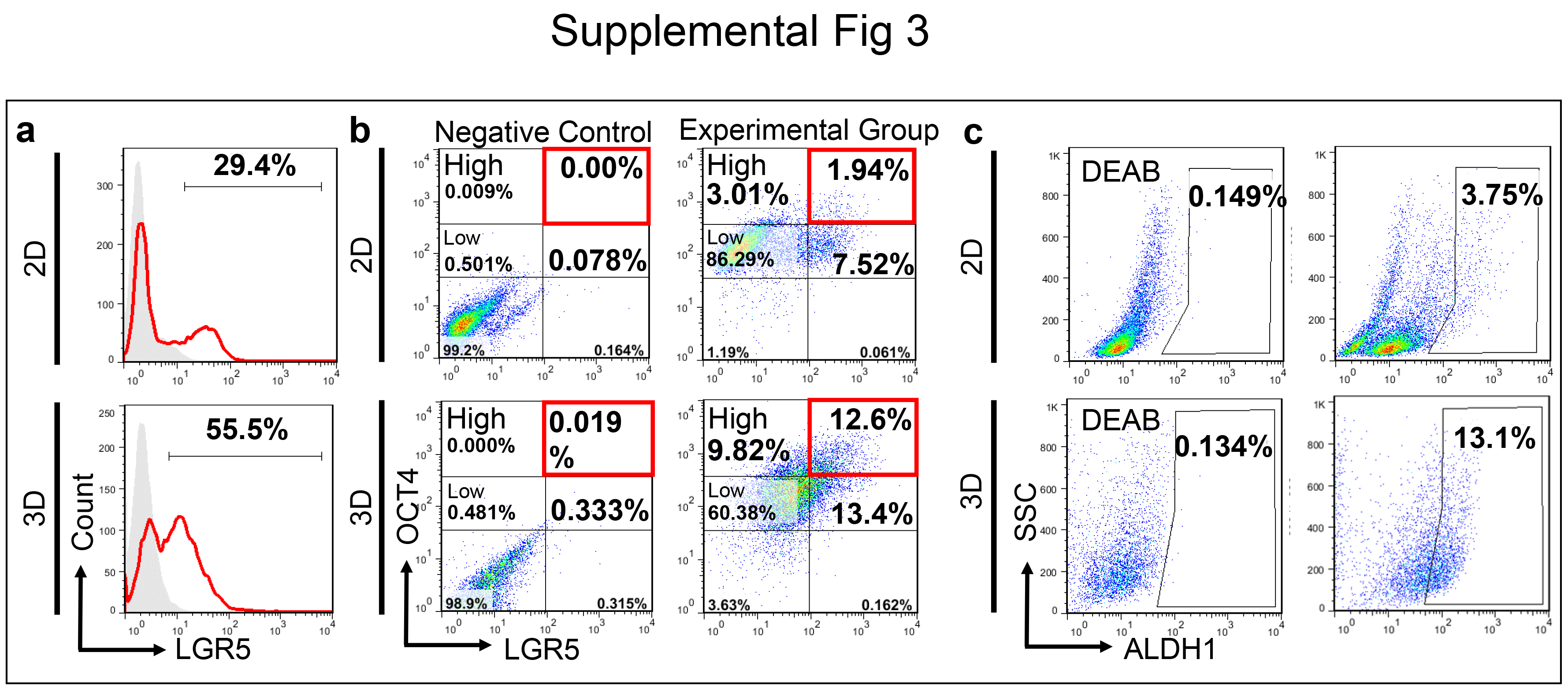

Supplement: Supplementary file 3 — Supplemental Fig 3 [file 41419_2020_2560_MOESM3_ESM.png]

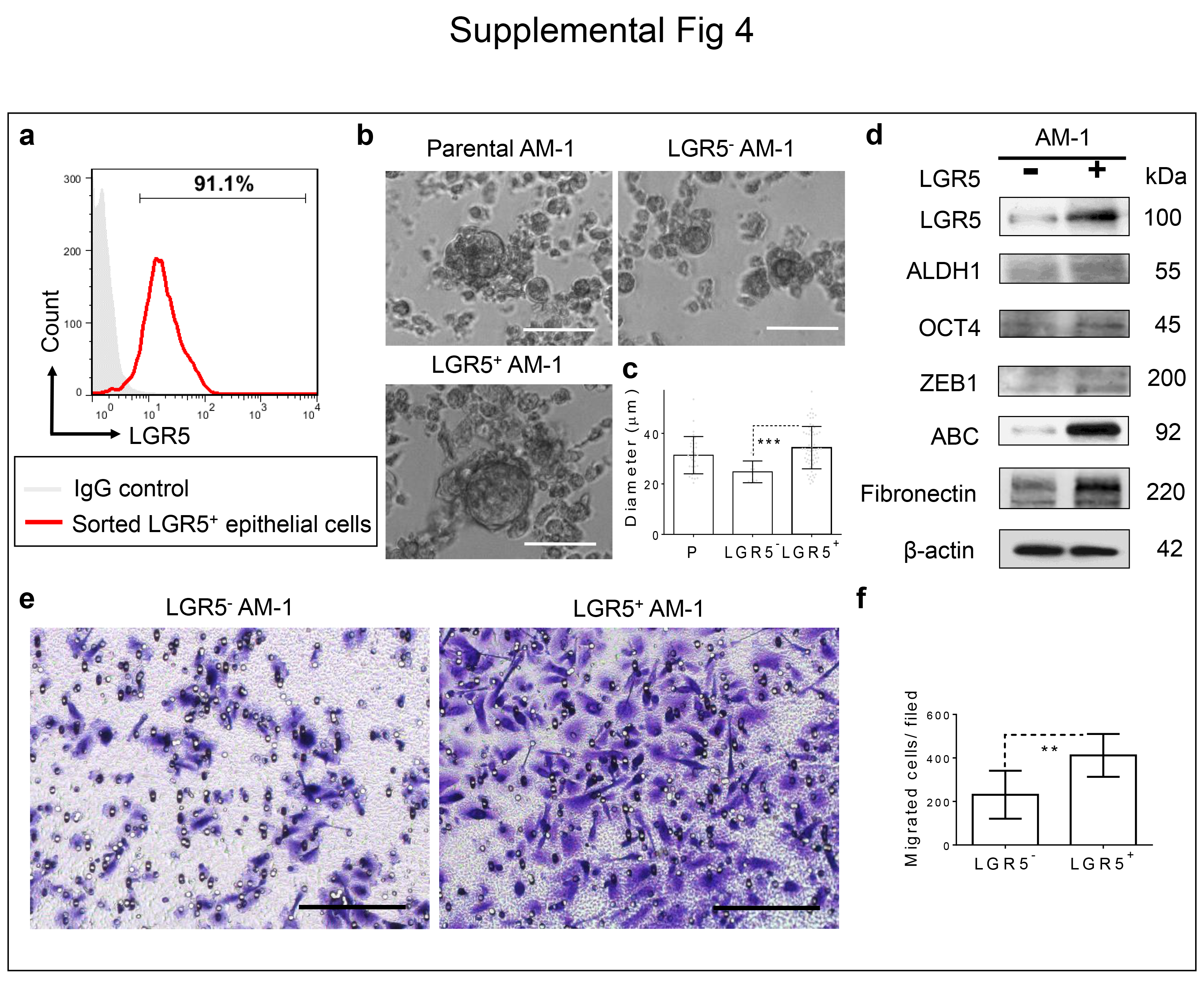

Supplement: Supplementary file 4 — Supplemental Fig 4 [file 41419_2020_2560_MOESM4_ESM.png]

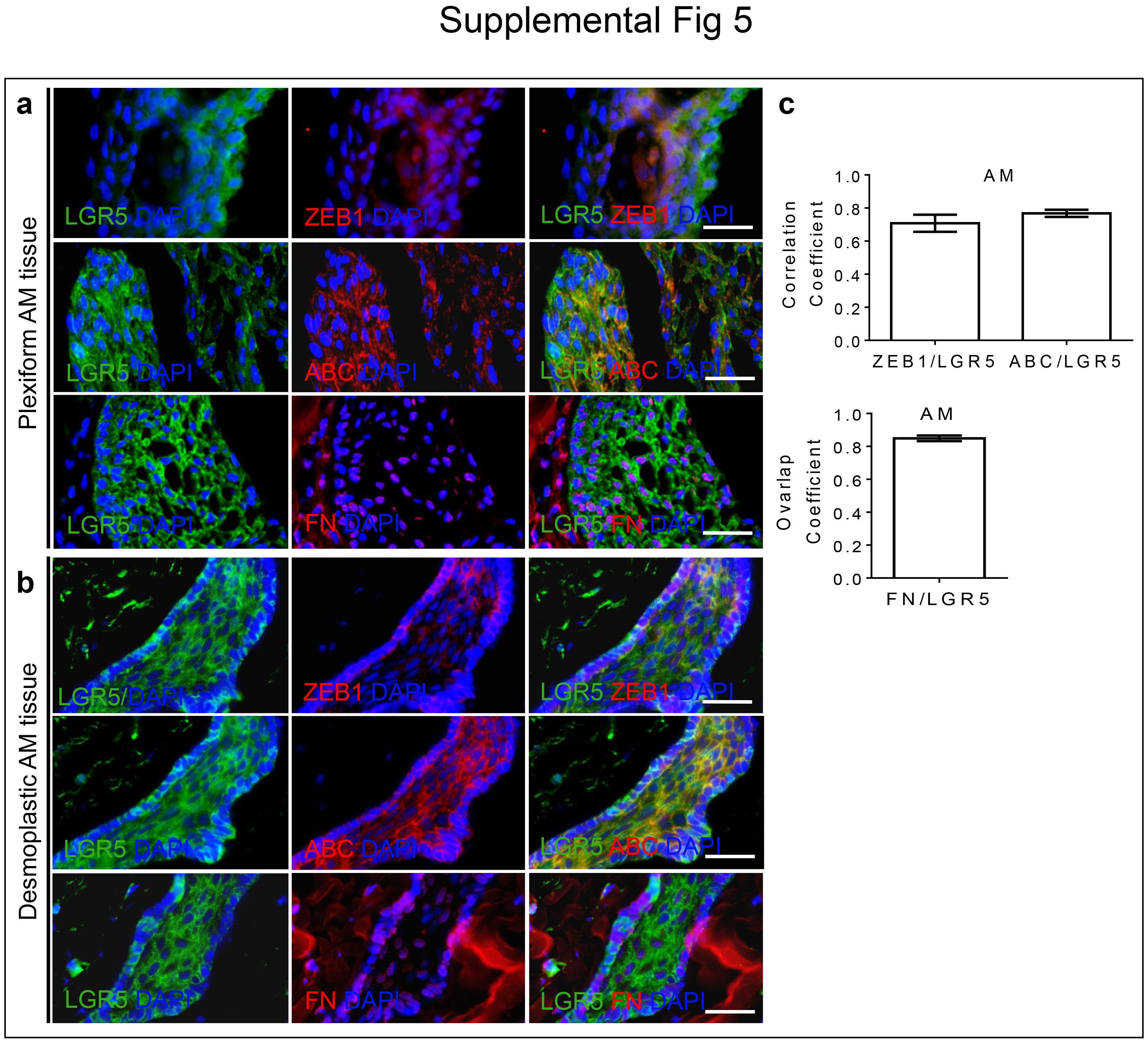

Supplement: Supplementary file 5 — Supplemental Fig 5 [file 41419_2020_2560_MOESM5_ESM.png]

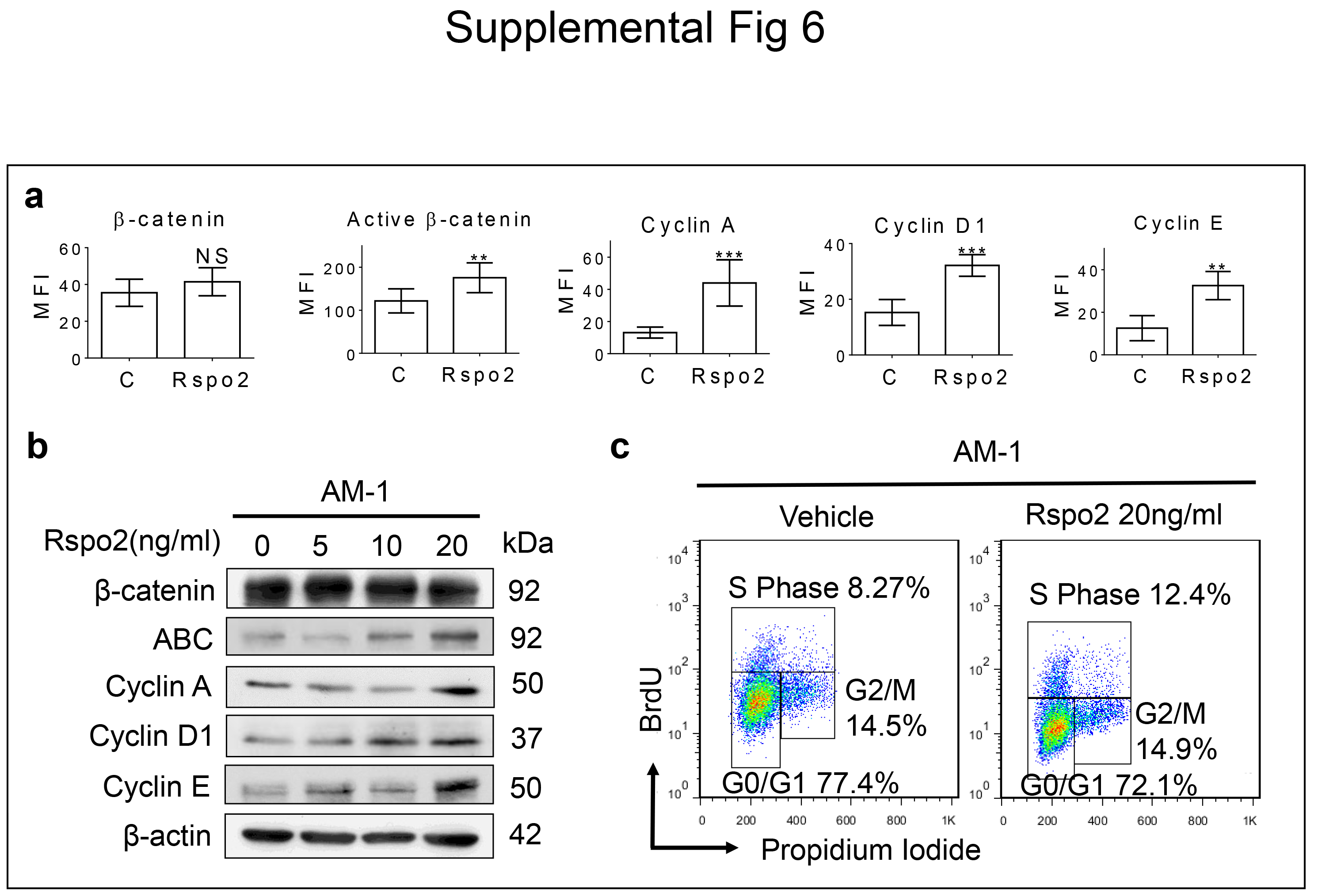

Supplement: Supplementary file 6 — Supplemental Fig 6 [file 41419_2020_2560_MOESM6_ESM.png]

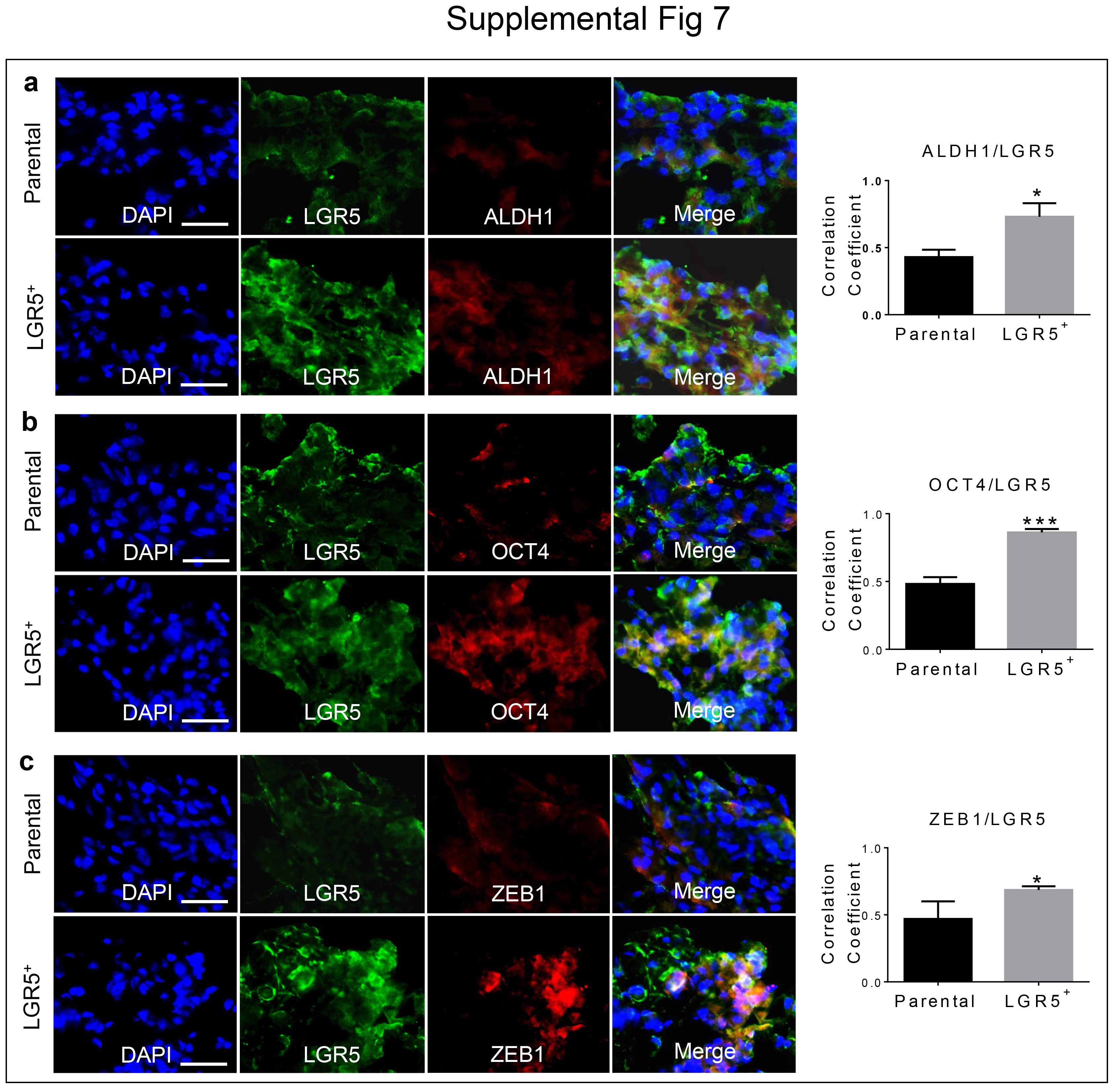

Supplement: Supplementary file 7 — Supplemental Fig 7 [file 41419_2020_2560_MOESM7_ESM.png]

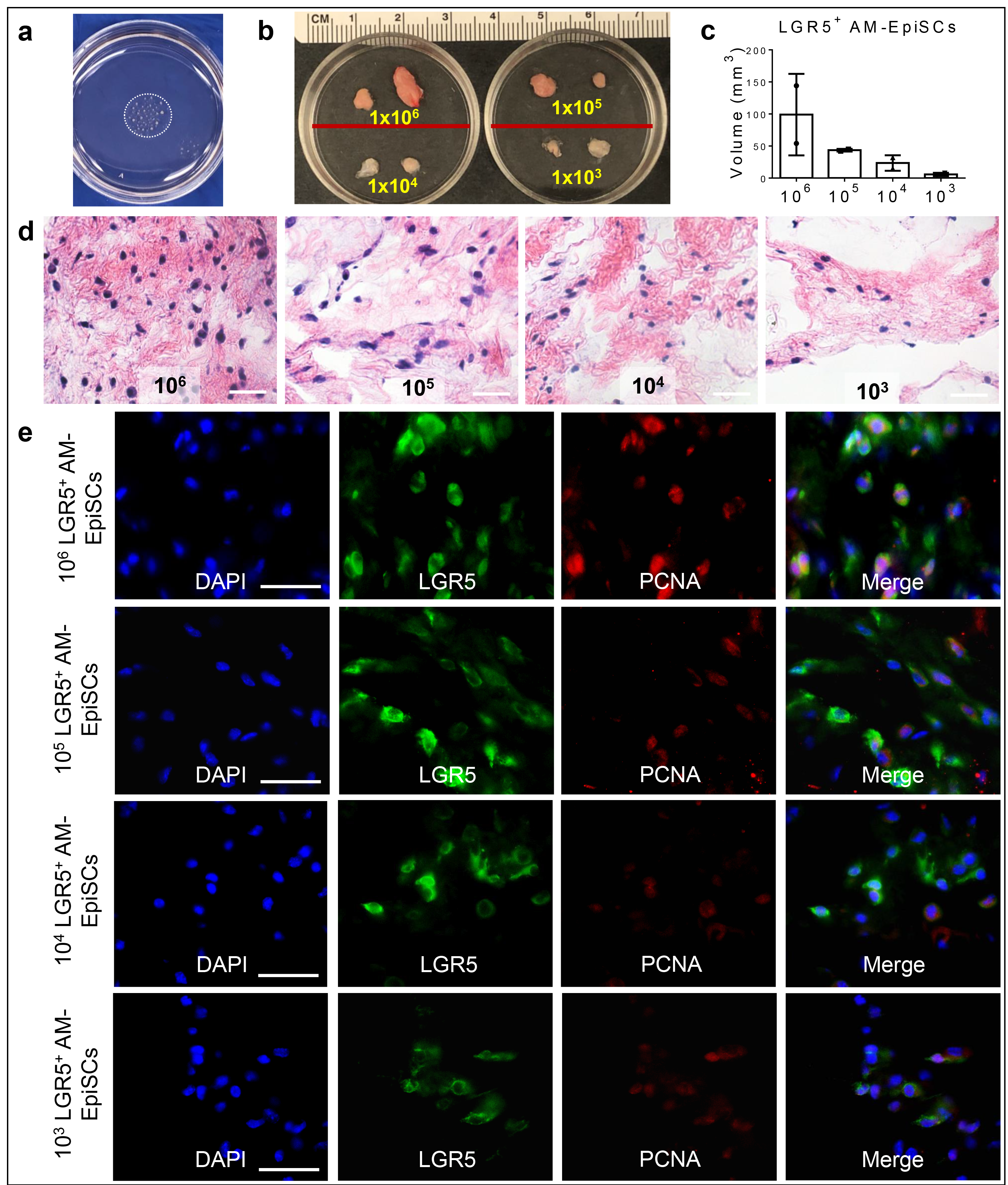

Supplement: Supplementary file 8 — Supplemental Fig 8 [file 41419_2020_2560_MOESM8_ESM.png]

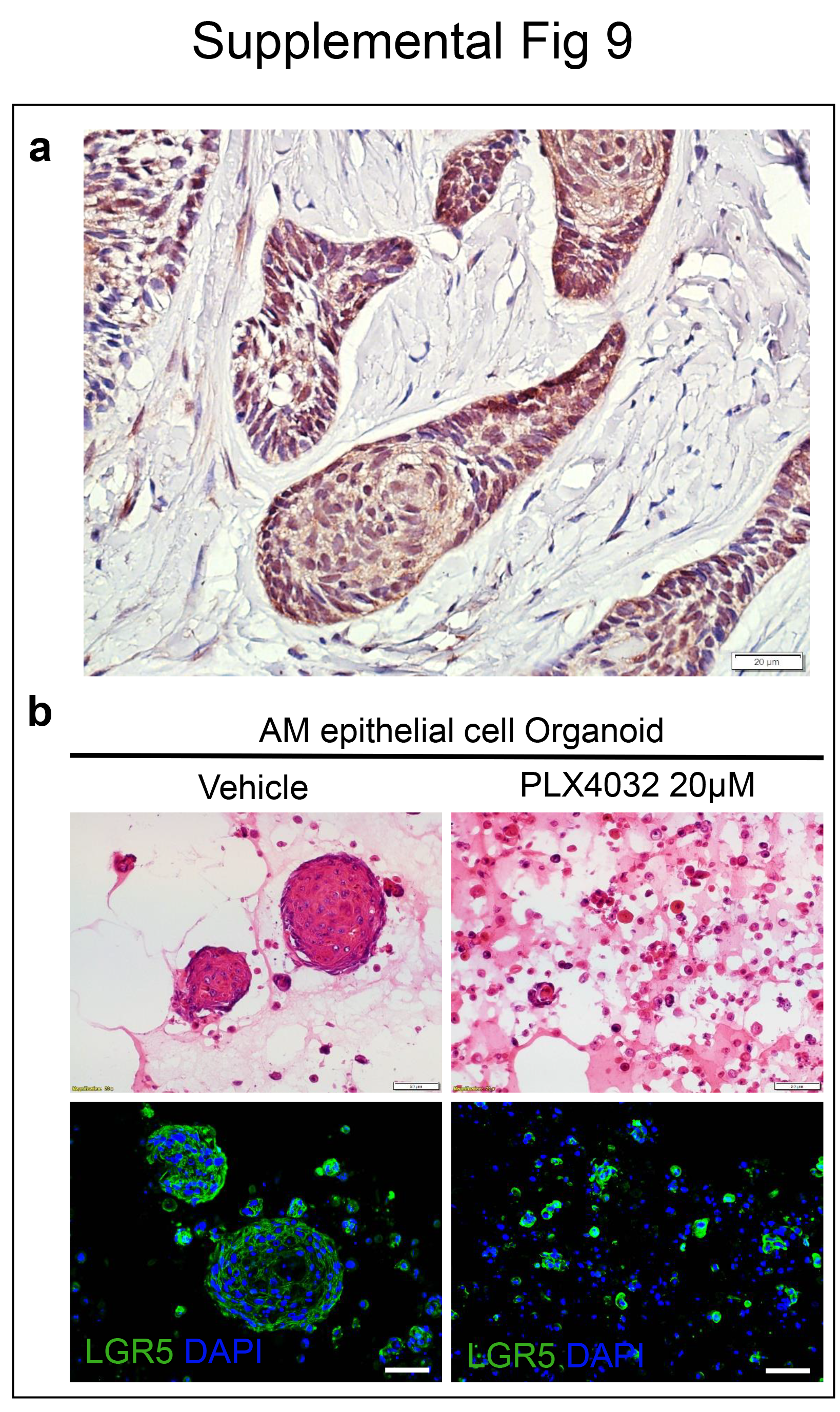

Supplement: Supplementary file 9 — Supplemental Fig 9 [file 41419_2020_2560_MOESM9_ESM.png]
