# Supplementary material for: LGR5+ epithelial tumor stem-like cells generate a 3D-organoid model for ameloblastoma
Source: Cell Death Dis. 2020 May 7;11(5):338. doi: 10.1038/s41419-020-2560-7 (PMC7206107; doi:10.1038/s41419-020-2560-7)
Supplement: Supplementary file 10 — Supplemental figure legends [file 41419_2020_2560_MOESM10_ESM.doc]

**Supplemental Fig 1.** **Expression of LGR5 in AM and odontogenic cysts (OC). a** Left, the quantification of H-score of LGR5 expression in stroma and epithelial islands of AM, respectively (n=15). Right, relative percentage was converted from the H-Score values. The immunoreactive signals of LGR5 expression are mainly localized in the AM epithelial islands, with an average 70.45% of LGR5^+^ cells in epithelial islands versus an average 18.62% of LGR5^+^ cells in stroma of the total fifteen AM tissues. The expression of LGR5 in AM epithelial islands was slightly higher in the plexiform (85.98%) than that in the follicular type (66.50%) (p< 0.05). H-Score of each sample was analyzed at least 5 different areas by Color Deconvolution of ImageJ software and data are mean ± SD. Two-tailed unpaired Student’s *t*-test. **p*<0.05. **b** The paraffin-embedded sections of OC were processed for IHC staining with a specific antibody for human LGR5 (n=6). Scale bars, 50μm. NAT: normal adjacent tissue (same patient).

**Supplemental Fig 2.** **Co-localization of LGR5 and certain stem cell-related genes in AM tissues**. **a** Dual-color immunofluorescence study showed that colocalization of Lgr5 and Pan cytokeratin (Pan-CK), ALDH1 and OCT4 in the plexiform tissue, respectively. Scale bars, 20μm. **b** Dual-color immunofluorescence study showed that colocalization of Lgr5 and Pan-CK, ALDH1 and OCT4 in the desmoplastic tissue, respectively. Scale bars, 20μm.

**Supplemental Fig 3.** **LGR5^+^ALDH1^+^OCT4^High^ are enriched in AM-1 cells under 3D spheroid-forming culture conditions**. **a** Flow cytometric analysis showed that LGR5^+^ cells were enriched by about two-fold (from 29.4% to 55.5%) in AM-1 cells under 3D-spheroid culture for 5 days. **b** The proportion of LGR5^+^OCT4^Low^, LGR5^+^OCT4^High^, and total OCT4^High^ cells in AM-1 cells cultured under 2D-monolayer culture and 3D-spheroid conditions was determined by flow cytometry. **c** The ALDH1 activity was increased by about three-fold (3.75% to 13.1%) in AM-1 cells under 3D-spheroid culture for 5 days. All results are representative of at least two to three independent experiments.

**Supplemental Fig 4.** **LGR5^+^ AM-1 cells exhibit self-renewal ability and EMT phenotypes.** **a** About 90% of sorted LGR5^+^ AM-EpiCs was positive for LGR5 as confirmed by flow cytometric analysis. **b** Sorted LGR5^+^ AM-1 formed larger 3D-spheroids than those by LGR5^-^ counterparts following cultured in 3D Matrigel for two weeks. Scale bars, 100μm. **c** The quantification of the results shown in **b**. Data are Mean ± SD (each group was measured 3 different random areas under the microscope), Two-tailed unpaired Student’s *t*-tests. ****p*<0.001. **d** Increased expression of stem cell-related genes, ALDH1 and OCT4, and EMT related genes/markers, ZEB1, active β-catenin (ABC) and fibronectin in sorted LGR5^+^ AM-1 cells in comparison to that in LGR5^-^ counterparts as determined by Western blot analysis. All results are representative of at least two to three independent experiments. **e**. The sorted LGR5^-^ and LGR5^+^ AM-1 (7x10^4^ cells/well in 200μl basal KBM2 medium) were seeded onto the upper chamber of 24-transwells and the lower chambers were filled with 600μl defined KGM-2 culture medium (n=3 for each group). After culture overnight (16h), the migrated cells were stained with crystal violet and images were taken under a microscope. Scale bars, 100μm. **f**. The quantification of the results shown in **e**. Data are Mean ± SD (each trans-well was measured 6 different random areas under the microscope with 100x magnification), Two-tailed unpaired Student’s *t*-tests. ***p*<0.01.

**Supplemental Fig 5**. **Co-localization of LGR5 and certain EMT-related gene expressions in AMs.** **a**, **b** Colocalization of LGR5 and specific EMT related genes/markers, ZEB1, ABC and fibronectin (FN) in the plexiform (**a**) and desmoplastic (**b**) AM tissues as determined by immunofluorescence studies. **c** The quantification of the results shown in **a**, **b**, and **Fig. 3f** (follicular type). N=3, each group was calculated at least three different areas by CellProfiler software and data are mean ± SD. Scale bars, 20μm. All results are representative of at least two to three independent experiments.

**Supplemental Fig 6.** **R-spondin stimulation promotes proliferation of AM epithelial cells.** **a** The quantification of the results from immunofluorescence studies shown in Fig. 3I. MFI: mean fluorescence intensity. Each group was measured 6 different random areas and data are mean ± SD. Two-tailed unpaired Student’s *t*-tests, NS=not significant, ***p*<0.01, ****p*<0.001. **b** The stimulation with Rspo2 for 48 h increased the expression of active β-catenin (ABC), cyclin A, D1 and E in AM-1 cells in a dose-dependent manner. **c** Flow cytometry showed increased proportion of AM-1 cells at S-phase after stimulation with Rspo2 (20ng/ml) for 48 h. All results are representative of at least two to three independent experiments.

**Supplemental Fig 7.** **LGR5^+^ AM epithelial cells exhibit propagating ability *in vivo*.** Following culturing in 3D Matrigel for three weeks, the *ex vivo* organoids formed by parental, sorted LGR5^+^ or LGR5^-^ AM epithelial cells were harvested and subcutaneously transplanted into the flank of nude mice. Two weeks post-transplantation, the tumor-like structures formed in nude mice were harvested for immunofluorescence studies. **a** Xenografted tumor-like structures formed by transplanted LGR5^+^ cells showed elevated co-expression of ALDH1 and LGR5 in comparison to those formed by transplanted parental cells. Scale bars, 20μm. Mean ± SD, two-tailed unpaired Student’s *t*-tests. **p*<0.05. **b** Xenografted tumor-like structures formed by transplanted LGR5^+^ cells showed elevated co-expression of OCT4 and LGR5 in comparison to those formed by transplanted parental cells. Scale bars, 20μm. Mean ± SD, two-tailed unpaired Student’s *t*-tests. ****p*<0.001. **c** Xenografted tumor-like structures formed by transplanted LGR5^+^ cells showed elevated co-expression of ZEB1 and LGR5 in comparison to those formed by transplanted parental cells. Scale bars, 20μm. Mean ± SD, two-tailed unpaired Student’s *t*-tests. **p*<0.05.

**Supplemental Fig 8.** **LGR5^+^ AM-EpiSCs exhibit self-renewal capability *in vivo*.** For cell dilution assay, we sorted LGR5^+^ cells were sorted from primary AM epithelial cells and then cultured in Matrigel (50μl) for two weeks at different cell numbers: 10^3^, 10^4^, 10^5^ and 10^6^ (n=2 each group). After two weeks, the organoids in Matrigel were transplanted subcutaneously into the dorsal skin of nude mice for one month. **a** The organoids in Matrigel. **b** Organoid xenografts were harvested one-month post-transplantation. **c** Calculation of the mean volume of organoid xenografts from different groups of animals. **d** Histological analysis of organoid xenografts by H & E staining. Sale bars, 20μm. **e** The expression of human LGR5 and proliferating cell nuclear antigen (PCNA) in organoid xenografts were determined by immunofluorescence study. Scale bars, 20μm.

**Supplemental Fig 9.** **LGR5^+^ AM-EpiSCs resist to the BRAF inhibitor.** **a** Immunohistochemistry study showed BRAF^V600E^ mutation in primary AM tissues. **b** 3D-organoids formed by AM-EpiCs for 4 days were treated with PLX4032 (20μM) for 6 days. Upper: PLX4032 interfered with 3D-organoid formation as determined by H&E staining. Lower: the residual PLX4032-resistant AM-EpiCs cells in 3D-organoids were positive for LGR5 as determined by immunofluorescence study. Scale bars, 20μm.
